# Glycan-Dependent Corneocyte Adherence of Staphylococcus epidermidis Mediated by the Lectin Subdomain of Aap

**DOI:** 10.1128/mBio.02908-20

**Published:** 2021-07-13

**Authors:** Paroma Roy, Alexander R. Horswill, Paul D. Fey

**Affiliations:** a Department of Pathology and Microbiology, University of Nebraska Medical Center, Omaha, Nebraska, USA; b Department of Immunology and Microbiology, University of Colorado School of Medicine, Aurora, Colorado, USA; University of Kansas; Harvard Medical School

**Keywords:** accumulation-associated protein, *Staphylococcus aureus*, *Staphylococcus epidermidis*, skin adherence

## Abstract

Staphylococcus epidermidis and other coagulase-negative staphylococci (CoNS) that colonize skin are known to promote skin immunity and inhibit colonization of pathogens that cause skin and soft tissue infections, including Staphylococcus aureus. However, S. epidermidis adherence to corneocytes, the cells that constitute the uppermost layer of the skin epidermis, remains poorly understood. Our study documents that S. epidermidis corneocyte adherence is dependent upon the accumulation-associated protein (Aap). Aap is composed of two distinct A and B domains. The A domain is comprised of a repeat region and a conserved L-type lectin domain, whereas the fibrillar B domain, which is comprised of G5 and E repeats, is linked to the cell wall in a sortase-dependent manner. Our studies revealed that adherence to corneocytes is dependent upon the lectin subdomain within the A domain. However, significant adherence was only observed when the lectin domain was expressed with both the A repeat and the B domain, suggesting further interactions between these three domains. Our data also suggest that the A repeat domain is important for stability or expression of Aap. Deglycosylation treatment suggested that glycans expressed in the host stratum corneum serve as potential binding partners for Aap-mediated corneocyte adherence. Last, bioinformatic analyses of the predominant commensal species of CoNS identified open reading frames (ORFs) homologous to *aap*, thus suggesting that Aap orthologues containing lectin-like domains may provide the basis for staphylococcal colonization of skin. Corroborating these observations, adherence to corneocytes in an S. aureus *mgrA* mutant was dependent upon SasG, the Aap orthologue in S. aureus.

## INTRODUCTION

The skin is our largest and outermost organ and consists of the epidermis, dermis, and subcutaneous tissue. A rich network of epithelial cells, lymphocytes, and antigen-presenting cells populate the epidermis and dermis, facilitating an immune response that is vital in wound healing and infection, as well as in modulating the microbiota that colonizes the skin (1). Despite the harsh physical landscape of skin, which is characterized by a desiccated, nutrient-poor, and acidic environment, this organ serves as a complex and dynamic ecosystem for millions of microorganisms. The skin is colonized by many bacterial genera, including Staphylococcus, Corynebacterium, Streptococcus, and Cutibacterium, with Staphylococcus epidermidis among the most well studied. Other frequent colonizing species of staphylococci include Staphylococcus hominis, Staphylococcus haemolyticus, Staphylococcus capitis, Staphylococcus lugdunensis, and Staphylococcus warneri, among 38 other identified species of coagulase-negative staphylococci (CoNS) (2–5). However, in certain contexts, including the state of immune activation or host genetic predisposition, skin microbial communities can be remodeled over time or in response to environmental challenges (6–8). Many common skin pathologies are associated with changes in the microbiota, and the state of dysbiosis may contribute to the disruption of immune homeostasis and increase disease symptomology.

Recent studies have documented that S. epidermidis and other CoNS function to protect against invading foreign pathogens, including Staphylococcus aureus, thus behaving as mutualists. Indeed, S. epidermidis has been shown to inhibit S. aureus nasal colonization and biofilm formation through production of the serine protease Esp (9) and subsequent degradation of S. aureus surface proteins and host receptors that are crucial for host-pathogen interactions (10). In a more recent study, it was shown that Staphylococcus lugdunensis inhibited S. aureus growth through the production of the cyclic peptide antibiotic lugdunin (11). Furthermore, S. epidermidis and S. hominis can secrete antimicrobial peptides (AMPs) that are able to synergize with the human cathelicidin AMP LL-37 to inhibit the growth of S. aureus. Strains producing these AMPs were depleted in individuals with atopic dermatitis (AD), who are frequently colonized with S. aureus. Moreover, the topical application of these antimicrobial-producing strains decreased the colonization of S. aureus in individuals with AD, demonstrating how dysbiosis of the skin microbiota can lead to disease and further documenting the importance of the beneficial CoNS strains (12). Last, the immune system has evolved closely with resident microorganisms in the skin to allow the maintenance of commensal partners and the elimination of possible pathogens. These skin-resident microbes are critical for establishing skin immune homeostasis. In particular, colonization with S. epidermidis has been shown to induce interleukin 17A-positive (IL-17A^+^) CD8^+^ T cells that localize to the epidermis and enhance innate barrier immunity and limit pathogen invasion. This process occurs through the coordinated action of skin-resident dendritic cell subsets and is not associated with inflammation, revealing that tissue-resident cells are poised to sense and respond to any alterations in microbial communities (13, 14).

Adhesion to host tissue is fundamental for bacteria to initiate a successful symbiosis (15). However, a mechanistic understanding of staphylococcal adherence to the skin surface remains poorly understood, and hence the molecular interactions existent at this bacterial-host interface is an area of significant interest. Adherence of S. aureus to corneocytes in patients with atopic dermatitis (AD) is promoted by the S. aureus surface protein ClfB, in addition to the synthesis of neutrophil extracellular traps (NETs) (16–18). However, AD is an altered skin condition that is complicated by mutations in filaggrin, leading to a loss of functional skin barrier and natural moisturizing factors (19). Indeed, S. aureus does not typically adhere to healthy skin, suggesting that ClfB ligands may be more accessible as a result of these pathophysiologic changes. The predominant commensal of the healthy skin, S. epidermidis, encodes fewer cell wall-anchored (CWA) surface proteins that function as adhesins (20), perhaps rendering it less able to compete for binding in the AD microenvironment. Many cell wall-anchored proteins of staphylococci are multifunctional, and distinct domains mediate adherence to different ligands and contribute to distinct phenotypes (21, 22). Among these, Aap is the best-studied adhesin factor and is found in approximately 85 to 95% of all S. epidermidis isolates, independent of culture source (23, 24). As reviewed by Gotz (25), Aap was first identified by Schumacher-Perdreau et al. in 1994 (26) and was further studied by Hussain and colleagues in 1997 (27). Mitomycin mutagenesis led to the isolation of a biofilm-negative mutant, M7, which lacked a 140-kDa exoprotein (26). Hussain and colleagues found an association between this 140-kDa protein and enhanced biofilm production, and thus it was referred to as the accumulation-associated protein (Aap) (27).

Several studies have demonstrated that Aap is a rod-like fibril extending from the cell that can be observed by electron microscopy (28–30). Bioinformatic analyses suggest that Aap is a multidomain protein that consists of two distinct domains, A and B (31). A similar domain arrangement also exists in SasG, the Aap orthologue in S. aureus (22). Following extracellular export directed by the N-terminal signal sequence, the protein is anchored to the cell wall by sortase via the C-terminal LPDTG motif (32). Two independent studies found that the A domain, which is composed of a repeat region and an L-type lectin domain, functions to facilitate adherence to abiotic surfaces (32, 33). Moreover, the N-terminal A domain is subjected to complete or partial proteolytic processing by the SepA metalloprotease (34, 35). This enables dimerization of B repeats on adjacent bacteria, thus facilitating intercellular adhesion and bacterial accumulation (29, 36, 37). The C-terminal B domain is composed of B repeats, each of which contains a G5 module and an E spacer region. Structural analysis supports a “zinc zipper” model whereby interaction between G5 domains is zinc-dependent (29, 30, 37).

A study conducted by Macintosh et al. investigating S. epidermidis colonization of the skin identified Aap as one of the adhesins with a potential function in corneocyte attachment (38). The heterologous expression of Aap on the surface of Lactococcus lactis increased corneocyte adhesion 30-fold, suggesting that Aap facilitates adhesion to corneocytes independent of other adhesins. Additionally, the A domain of Aap was implicated in mediating this function, as the recombinant A domain of Aap partially blocked corneocyte binding by S. epidermidis NCTC 11047 and other *aap*-positive S. epidermidis clinical isolates. Moreover, another adhesin, serine-aspartate repeat-containing protein F (SdrF), has also been shown to bind recombinant proteins keratin 1 and 10, normal human epidermal keratinocytes (NHEKs), and human nasal epithelial cells (HNECs), suggesting a potential function in skin adherence (39).

In this study, we used isogenic mutants to document that S. epidermidis Aap mediates adherence to healthy skin. Furthermore, we constructed S. epidermidis strains expressing various Aap domains, and, in conjunction with blocking studies, documented that adherence to corneocytes is dependent upon the lectin subdomain within the A domain of Aap. Furthermore, we document that Aap-dependent adherence is significantly reduced following enzymatic cleavage of specific glycan structures present on the corneocytes of healthy skin. Last, we provide evidence of Aap orthologues in other staphylococcal species and document that S. aureus adheres to healthy skin in an SasG-dependent manner when *sasG* transcription is derepressed via an *mgrA* mutation.

## RESULTS

### Identification of the S. epidermidis factor responsible for adherence to corneocytes.

**FIG 1:**
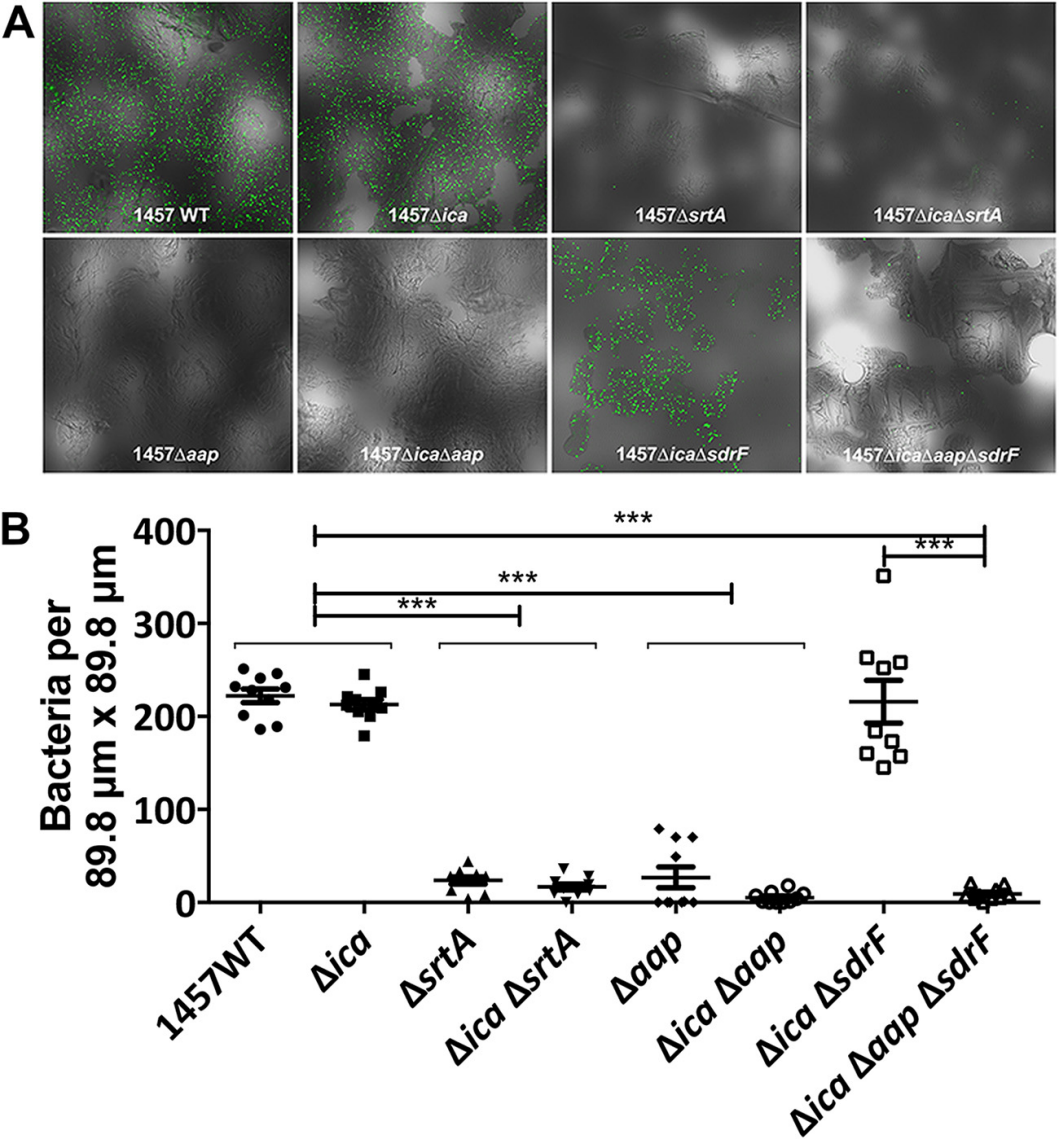
Aap facilitates adherence of Staphylococcus epidermidis to corneocytes. (A) Sections (353.6- μm × 353.6-μm) of the corneocyte adherence disks observed by confocal microscopy at 40 × 0.6 magnification. Each disk was incubated with green fluorescent protein (GFP) expressing 1457 and mutant strains for 45 min at 37°C to analyze the number of adherent bacteria after washing. (B) Mean numbers (with standard error of the mean [SEM]) of adherent 1457/pCM29 and mutants attached to corneocytes (per 89.8-μm × 89.8-μm section) as shown in panel A. Comparisons between individual mutants were performed by two-tailed unpaired Student’s *t* test with 95% confidence intervals. ***, *P* < 0.0001.

To identify the factor(s) responsible for S. epidermidis adherence to skin, a corneocyte binding assay was developed and performed with an optical density (OD)-adjusted suspension of different S. epidermidis 1457 strains that constitutively express green fluorescent protein (GFP). Preliminary studies suggested that an OD at 600 nm (OD_600_) of 0.15 (∼10^7^ CFU/ml) facilitated enumeration of adherent S. epidermidis cells (see Fig. S1 in the supplemental material). Since enhanced polysaccharide intercellular adhesin (PIA) production is a hallmark of strain 1457 (40), the function of PIA in skin adhesion was assessed using the 1457 and 1457 Δ*ica* strains. These experiments documented that binding of both 1457 and 1457 Δ*ica* strains to the corneocyte-adhered discs was similar, as assessed by confocal microscopy (Fig. 1A and B). Thus, this finding suggests that S. epidermidis skin adhesion is independent of PIA and is consistent with the hypothesis that the primary bacterial attachment on the skin is a protein-dependent mechanism.

10.1128/mBio.02908-20.4 FIG S1 Corneocyte adherence assay. (A) Representative image of S. epidermidis 1457/pCM29 adhered to human corneocytes after washing, when incubated with bacterial suspensions at an OD_600_ of 0.15, 1.0, or 1.5. Magnification, 40 × 0.6. (B) Magnification, 63 × 1.5. Download FIG S1, TIF file, 0.8 MB. Copyright © 2021 Roy et al. 2021 Roy et al. https://creativecommons.org/licenses/by/4.0/ This content is distributed under the terms of the Creative Commons Attribution 4.0 International license.

Sortase mediates the covalent binding of cell surface proteins containing an LPXTG motif to peptidoglycan (41). Indeed, inactivation of *srtA* reduces the localization of cell wall-anchored (CWA) proteins to the cell surface of S. epidermidis (32). To investigate the involvement of LPXTG-anchored surface protein adhesins in binding to the skin, a 1457 Δ*srtA* mutant was constructed and investigated for its corneocyte binding efficiency. Both the 1457 Δ*srtA* and 1457 Δ*ica* Δ*srtA* mutants displayed negligible binding to corneocytes (Fig. 1A and B), suggesting the involvement of a CWA protein in the skin adhesion phenotype. S. epidermidis expresses 11 proteins containing an LPXTG motif (31), of which Aap and SdrF have been previously shown to possess adhesive properties and have been implicated in facilitation of S. epidermidis colonization of skin (38, 39). Similarly to our findings with the sortase mutant, corneocyte discs incubated with Aap-negative S. epidermidis 1457 Δ*aap* and 1457 Δ*ica* Δ*aap* mutants showed negligible adherence to corneocytes, in contrast to the Aap-positive 1457 and 1457 Δ*ica* mutant strains (Fig. 1A and B), suggesting that the CWA protein Aap mediates bacterium-host interaction.

To test the function of SdrF in aiding Aap-dependent binding of 1457 to corneocytes, an *sdrF* allelic replacement mutant was constructed in the 1457 Δ*ica* mutant background. The corneocyte binding assay revealed that adhesion of the 1457 Δ*ica* Δ*sdrF* mutant was similar to that of the 1457 Δ*ica* mutant (Fig. 1A and B), implying that in our assay, SdrF does not function to facilitate adherence to corneocytes. Furthermore, the finding that the 1457 Δ*ica* Δ*aap* Δ*sdrF* mutant phenocopied the 1457 Δ*ica* Δ*aap* mutant rather than the 1457 Δ*ica* Δ*sdrF* mutant (Fig. 1A and B) strongly supports the importance of Aap in S. epidermidis skin colonization, irrespective of the presence of other CWA adhesins. A defective adherence phenotype of the *aap* mutation also diminishes the likelihood of two or more CWA proteins contributing collectively to the adhesive properties in this strain.

### Identification of the Aap domain that mediates binding of S. epidermidis to corneocytes.

**FIG 2:**
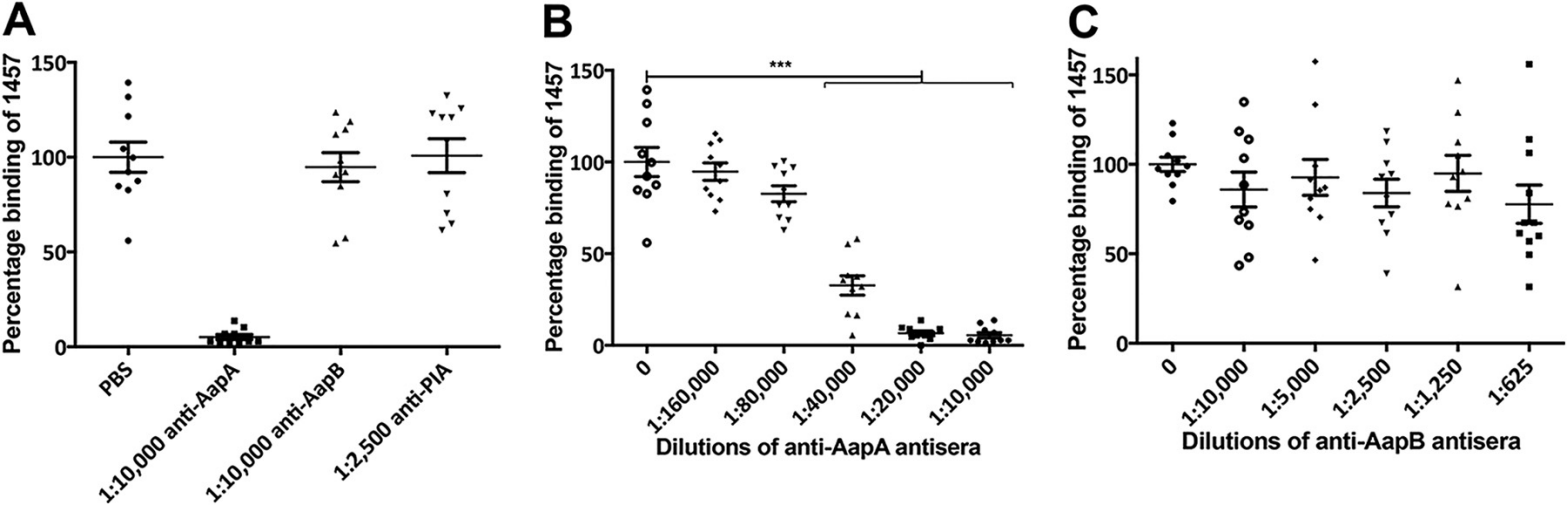
Relative blocking of 1457/pCM29 using anti-AapA or anti-AapB antisera. (A) Anti-AapA antiserum blocked adherence, whereas anti-AapB antiserum did not. Anti-polysaccharide intercellular adhesin (PIA) antibody was used as a negative control. (B) Dose-dependent decrease of adherent bacteria with increasing concentration of anti-AapA antiserum. (C) No significant decrease in adherence was observed with increasing concentration of anti-AapB antiserum. The number of adherent 1457/pCM29 bacteria preincubated with the indicated dilutions (concentrations) of anti-AapA or anti-AapB antisera was normalized to the mean number of adherent 1457/pCM29 bacteria preincubated with PBS only (considered 100). Comparisons were performed by two-tailed unpaired Student’s *t* test with 95% confidence intervals. ***, *P* < 0.0001.

Aap consists of structurally distinct domains (33). The N-terminal A domain of Aap, as well as its S. aureus orthologue SasG, have been previously implicated in promoting adhesion to mammalian cells and artificial surfaces (33, 38, 42). Therefore, we hypothesized that the Aap-mediated adherence of strain 1457 to corneocytes observed in Fig. 1 is facilitated by the A domain. To test this hypothesis, a bacterial blocking assay was performed using antiserum raised against the A or B domain of Aap (23). As shown in Fig. 2A, anti-AapA antiserum blocked adherence of 1457 to corneocytes, whereas anti-AapB antiserum did not, suggesting that Aap-mediated adherence of S. epidermidis 1457 to corneocytes from the skin primarily involves the A domain. A dose-dependent decrease of adherent bacteria was also observed with increasing concentration of anti-AapA antiserum (Fig. 2B), confirming the function of the Aap A domain in the facilitation of 1457 binding to corneocytes. In contrast, increasing the concentration of anti-AapB antiserum had no effect on adherence to corneocytes (Fig. 2C). Last, antibody raised against PIA (a noncontributing factor in bacterium-skin adherence) was used as a negative control and had no effect on the binding of bacteria (Fig. 2A). Therefore, the 1457 Δ*ica* strain was subsequently used as the wild-type strain background for further studies.

**FIG 3:**
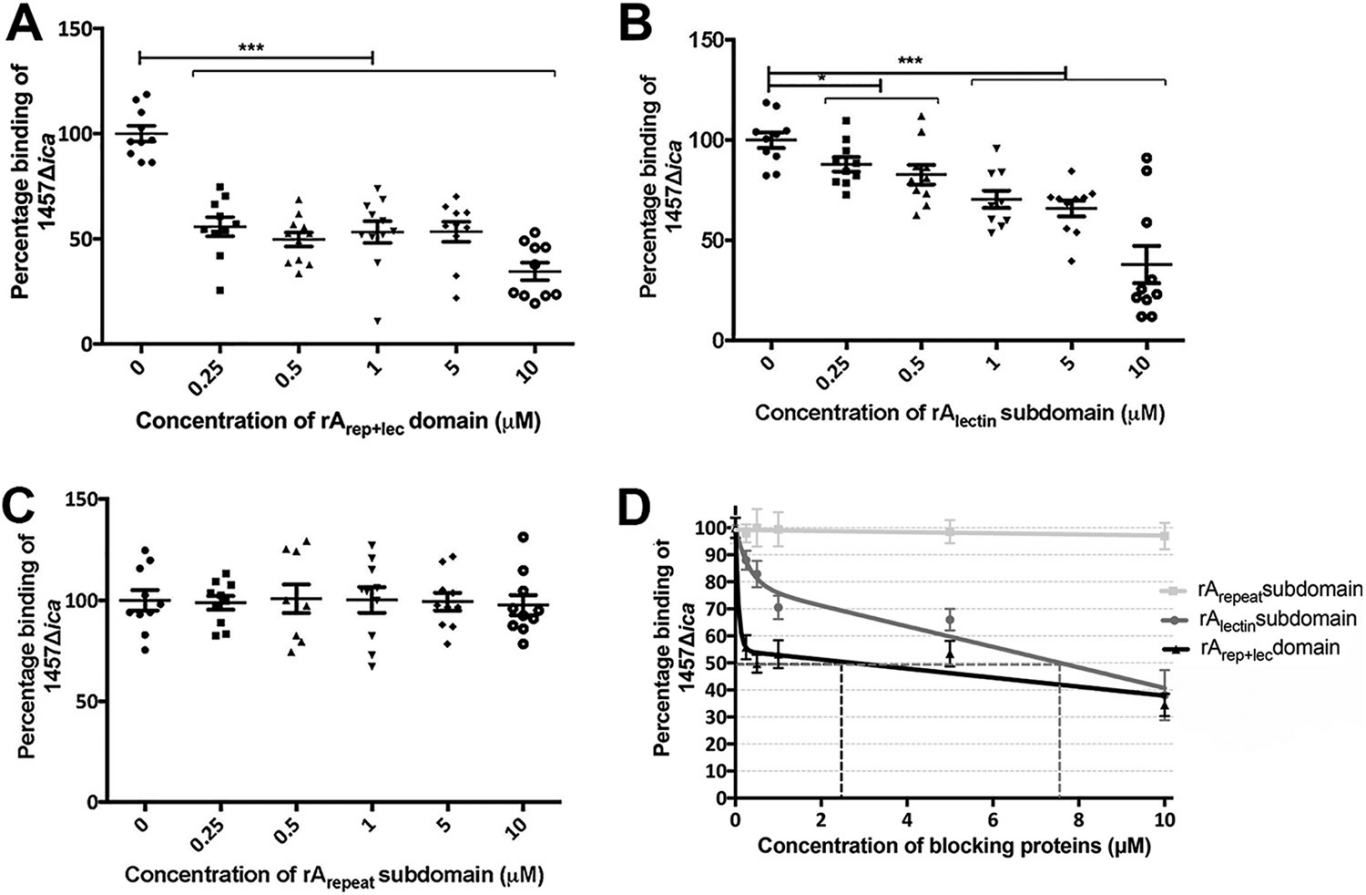
Relative blocking of S. epidermidis by recombinant Aap A subdomains. Effect of rAapA subdomains (A repeat and/or lectin) on S. epidermidis binding to corneocytes. (A) The rA_rep+lec_ domain and (B) the rA_lectin_ subdomain block the binding of 1457 Δ*ica*/pCM29 to corneocytes. In contrast, (C) the rA_repeat_ subdomain was unable to block adherence. (D) Normalization of the blocking with recombinant Aap A domain and its subdomain proteins. The number of adherent 1457 Δ*ica*/pCM29 bacteria after blocking the corneocytes with the indicated concentrations of the above-mentioned recombinant proteins was normalized to the mean number of 1457 Δ*ica*/pCM29 bacteria adherent to corneocytes blocked with PBS only (considered 100). Comparisons were performed by two-tailed unpaired Student’s *t* test with 95% confidence intervals. *, *P* < 0.05; ***, *P* < 0.0001.

The A domain is composed of a conserved 222-amino-acid globular subdomain that is predicted by bioinformatic analysis to be an L-type lectin-like domain. This lectin-like subdomain is positioned C-terminally to a strain-dependent, variable number of imperfect 16-amino-acid repeat regions (33). However, the particular subdomain that facilitates binding to corneocytes is yet to be identified. To further determine the contribution of the two subdomains, the ability of recombinantly expressed and purified rA_repeat_ and rA_lectin_ subdomain proteins to block adhesion was tested and compared to that of the complete rA_rep+lec_ domain (Fig. 3A to C). These data demonstrate that the rA_lectin_ subdomain blocked the binding of 1457 to corneocytes in a concentration-dependent manner, up to a maximum of 60% at 10 μM, similarly to the rA_rep+lec_ domain (Fig. 3D). In contrast, the rA_repeat_ subdomain was unable to block adherence. These results suggest that the L-type lectin-like subdomain is the key subdomain that contributes to the Aap A domain-mediated bacterium-host attachment.

### Binding of 1457 Δ*ica* isogenic mutants expressing truncated variants of Aap to corneocytes.

**FIG 4:**
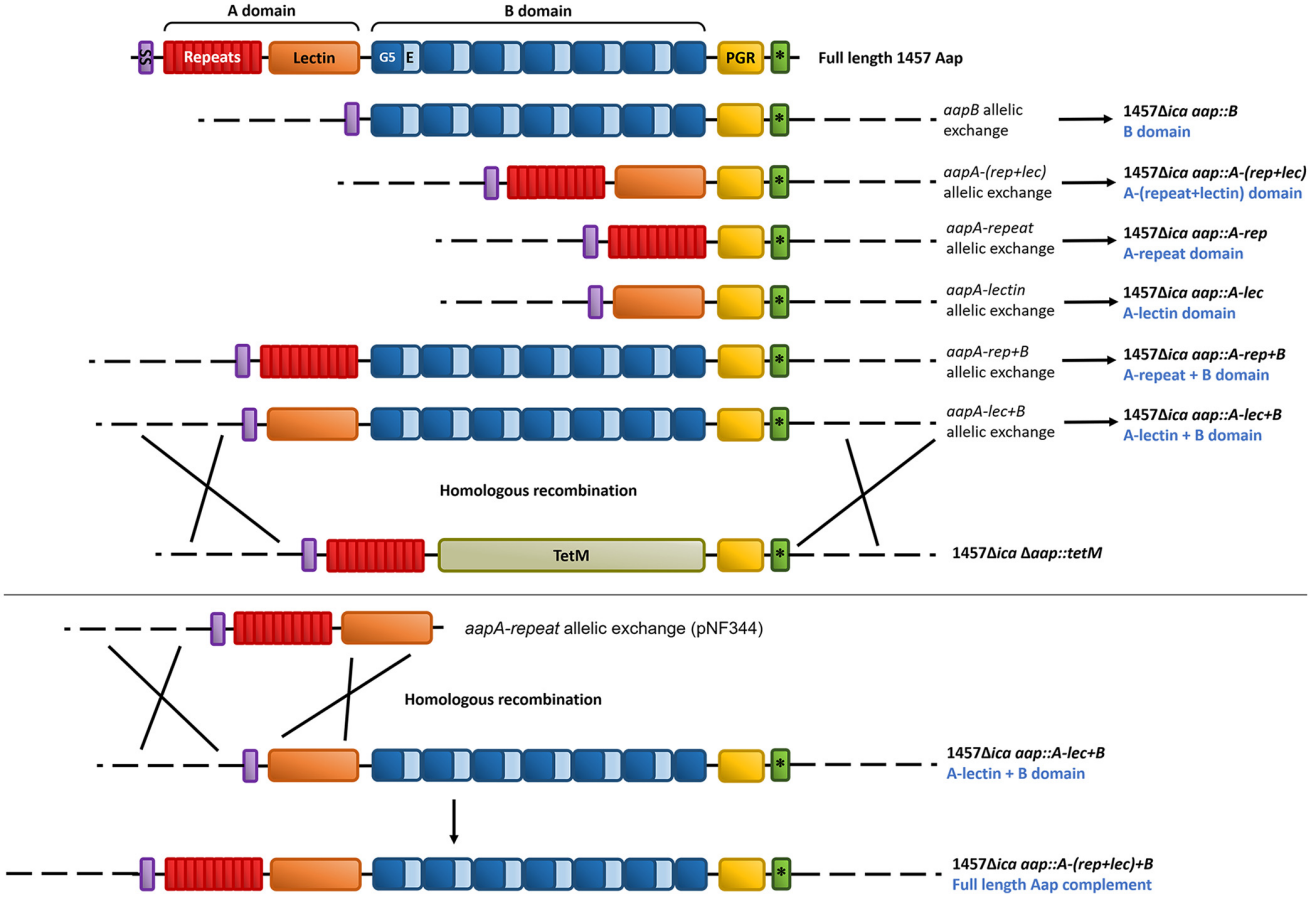
Construction of mutants expressing specific domains of Aap. Allelic replacement vectors were constructed to delete specific regions of Aap. Note that intact Aap was reconstructed via insertion of the A-repeat domain into the 1457 *icaADBC*::*dhfr aap*::*A-lec+B* construct.

Isogenic mutants expressing truncated variants of Aap were generated to confirm the significance of the individual domains of Aap in adherence to corneocytes. Constructs expressing different combinations of the A and B domains were inserted via allelic replacement into the *aap*::*tetM* allele of the 1457 *ica*::*dhfr aap*::*tetM* strain, thus replacing *aap*::*tetM* with functional *aap-*expressing specific domains. All constructs contained the native *aap* promoter and included the N-terminal signal sequence for extracellular export of the translated protein. The proline/glycine-rich region (PGR) domain, which has been shown to form an extended stalk to resist compaction (43), was added, in addition to the C-terminal LPDTG sortase recognition motif for anchoring the exported protein to the cell wall. The generated knock-in mutants are shown in Fig. 4; pCM29 expressing GFP was transduced into each constructed mutant. To minimize proteolytic cleavage of Aap, the above mutants were grown in tryptic soy broth (TSB) containing the protease inhibitor α_2_-macroglobulin.

**FIG 5:**
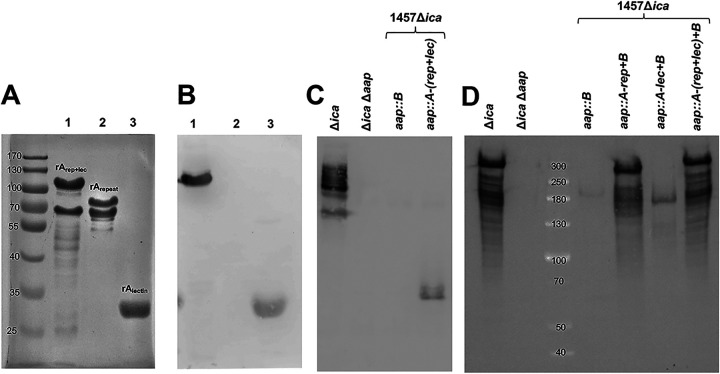
Use of anti-AapA and anti-AapB to assess expression of Aap in truncated variants. (A) The recombinant A_rep+lec_ domain (upper band) (lane 1), the rA_repeat_ subdomain (lane 2), and the rA_lectin_ subdomain (lane 3) were electrophoresed through a 10% SDS-PAGE gel and stained with Coomassie blue. Two bands were visible for both the rA_rep+lec_ domain and the rA_repeat_ subdomain due to the included metalloprotease cleavage site, which is present within the A domain at Leu 335, between the A-repeat and the A-lectin subdomains. Liquid chromatography-tandem mass spectrometry (LC-MS/MS) analysis of the upper and lower bands of the rA_rep+lec_ domain indicated that the lower band protein is truncated at the C terminus, comprising essentially the A-repeat subdomain. Note that the rA_rep+lec_ domain and the rA_repeat_ subdomain had aberrant migration. The predicted molecular weights of the rA_rep+lec_ domain, the rA_repeat_ subdomain, and the rA_lectin_ subdomain are 57 kDa, 32 kDa, and 25 kDa. (B) A Western blot analysis was performed using anti-AapA antibody to detect recombinant proteins. Note the lack of detection of the rA_repeat_ subdomain in lane 2 and the lower band in lane 1. (C) Cell wall proteins were isolated from the 1457 Δ*ica*, 1457 Δ*ica* Δ*aap*, 1457 Δ*ica aap*::*B*, and 1457 Δ*ica aap*::*A-*(*rep+lec*) strains and electrophoresed through a 7% SDS-PAGE gel. Aap expression in each mutant was subsequently detected using anti-AapA antibody via Western blot analysis. (D) Cell wall proteins were isolated from the 1457 Δ*ica*, 1457 Δ*ica* Δ*aap*, 1457 Δ*ica aap*::*B*, 1457 Δ*ica aap*::*A-rep+B*, 1457 Δ*ica aap*::*A-lec+B*, and 1457 Δ*ica aap*::*A-*(*rep+lec*)*+B* strains and electrophoresed through a 7% SDS-PAGE gel. Aap expression in each mutant was subsequently detected using anti-AapB antibody via Western blot analysis.

To confirm that the constructed knock-in mutants were able to express the appropriate Aap domains on the cell wall, Western blot analysis was performed using anti-AapA and anti-AapB antibodies. First, to determine if the polyclonal anti-AapA antibody could recognize the A_repeat_ and A_lectin_ subdomains individually, a Western blot analysis was performed using purified rA_rep+lec_ domain and rA_repeat_ and rA_lectin_ subdomains. Although anti-AapA antibody could readily detect the rA_rep+lec_ domain, the rA_repeat_ subdomain was not detected, and the rA_lectin_ subdomain was weakly detected (Fig. 5A and B). Therefore, we were unable to confirm if the 1457 Δ*ica aap*::*A-rep* strain expressed the A repeat domain, due to the lack of antibody recognition when Western blot analysis was performed on its cell wall proteins using anti-AapA antibody (data not shown). Furthermore, we were unable to detect expression of the Aap A-lectin domain from the isolated cell wall proteins of the 1457 Δ*ica aap*::*A-lec* strain (data not shown). However, using anti-AapA antibody, we were able to detect the Aap A domain from the 1457 Δ*ica aap*::*A-*(*rep+lec*) strain (Fig. 5C). Last, cell wall proteins were isolated from the isogenic mutants expressing truncated Aap containing the B domain, and a Western blot analysis was performed using anti-AapB antibody (Fig. 5D). We found that anti-AapB antibody readily detected Aap from the 1457 Δ*ica* and 1457 Δ*ica aap*::*A-rep+B* strains. However, Aap was weakly detected in the 1457 Δ*ica aap*::*B* and 1457 Δ*ica aap*::*A-lec+B* strains. Interestingly, genetic insertion of the A repeat sequence of *aap* into the 1457 Δ*ica aap*::*A-lec+B* strain, generating the 1457 Δ*ica aap*::*A*-(*rep+lec*)*+B* strain, restored expression of Aap to the levels observed in the 1457 Δ*ica* and 1457 Δ*ica aap*::*A-rep+B* strains. These data may suggest that the A domain, and the A repeat region in particular, may be required for stability of Aap. Importantly, it is well known that Aap does not migrate according to size in SDS-polyacrylamide gels, presumably due to the PGR repeats (43). Interestingly, the ∼180-kDa band detected in the 1457 Δ*ica aap*::*B* strain was slightly larger in size than that detected in the 1457 Δ*ica aap*::*A-lec+B* strain, which was not predicted (Fig. 5D). Additionally, it is important to note that the presence of the Aap A repeat subdomain also resulted in aberrant migration of the rA_rep+lec_ domain and rA_repeat_ subdomain during SDS-PAGE, during which both migrated to a larger size than predicted (Fig. 5A). Accordingly, the major band detected near 300 kDa in the 1457 Δ*ica* and 1457 Δ*ica aap*::*A-*(*rep+lec*)*+B* strains was slightly larger in size than those in the 1457 Δ*ica aap*::*A-rep+B* strain (Fig. 5D). However, the difference in size between the major bands of the 1457 Δ*ica aap*::*A-rep+B* and 1457 Δ*ica aap*::*A-lec+B* strains was much greater than predicted (Fig. 5D).

**FIG 6:**
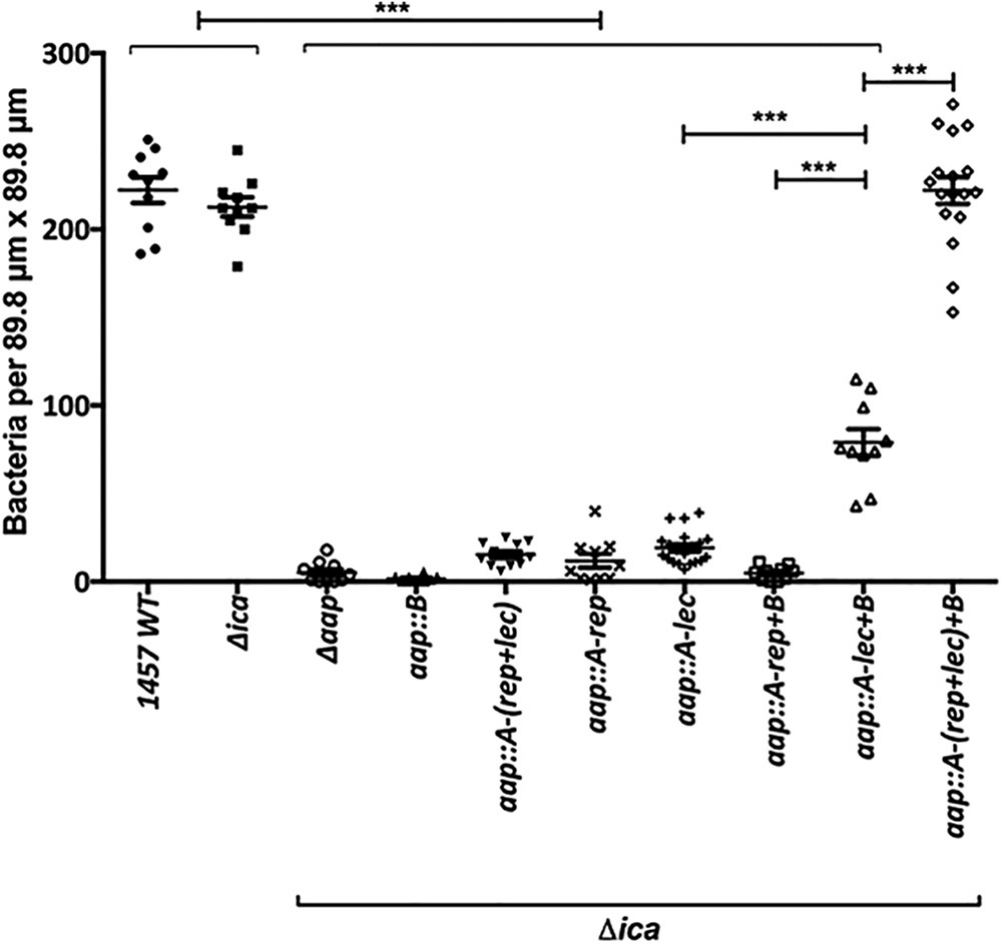
Corneocyte binding of 1457 Δ*ica* isogenic mutants expressing truncated variants of Aap. Mean number (with SEM) of adherent 1457 Δ*ica* truncated *aap* variants expressing GFP (per 89.8-μm × 89.8-μm sections). Comparisons between individual mutants were performed by two-tailed unpaired Student’s *t* test with 95% confidence intervals. ***, *P* < 0.0001.

Our blocking data suggested that the A domain, and, in particular, the lectin subdomain, was required to mediate adherence to corneocytes. To further confirm the contribution of the A repeat region and the A lectin subdomain, the corneocyte binding assay was performed with the 1457 Δ*ica* strain expressing only certain domains of Aap (Fig. 6). We found that even though the expression of Aap in the 1457 Δ*ica aap*::*A-rep+B* strain was comparable to that in the 1457 Δ*ica* strain (Fig. 5D), no corneocyte adherence was detected in the 1457 Δ*ica aap*::*A-rep+B* strain (Fig. 6). In contrast, even though the expression of Aap in the 1457 Δ*ica aap*::*A-lec+B* strain was reduced in comparison to that in the 1457 Δ*ica aap*::*A-rep+B* strain (Fig. 5D), significant corneocyte adherence was observed for the 1457 Δ*ica aap*::*A-lec+B* strain (Fig. 6). These data confirm that the lectin subunit of the A domain, and not the A repeat region, is responsible for corneocyte adherence. The corneocyte binding ability of the 1457 Δ*ica aa*p::*A*-(*rep+lec*)*+B* strain, expressing the B domain with the full-length A domain, was restored to the level of the 1457 and 1457 Δ*ica* strains (Fig. 6). These results suggest that the A-repeat domain may function to properly position the lectin subdomain for appropriate interaction with the corneocyte ligand in addition to the aforementioned possibility of conferring expression stability, as see in Fig. 5D. Furthermore, negligible corneocyte adherence was detected using the 1457 Δ*ica aap*::*A*-(*rep+lec*) strain, suggesting that the B domain may function to extend the A domain away from the cell wall to bind a ligand (Fig. 6). Additionally, no corneocyte adherence was observed for the 1457 Δ*ica aap*::*A-lec*,1457 Δ*ica aap*::*A-rep*, and 1457 Δ*ica aap*::*B* strains (Fig. 6).

### Glycan distribution on the surface of corneocytes.

L-type lectin domains are protein domains with homology to the leguminous plant lectins which specifically bind carbohydrates. Therefore, we hypothesized that the predicted lectin domain of Aap has affinity for glycan moieties in the stratum corneum (SC) corneocytes that serve as binding partners. Eukaryotic cells have a rich and diverse surface glycan coat that constitutes the cell interface with the environment (44). However, the glycome (the sum of its diverse glycan structures) on every human tissue is distinct and is defined by expression levels of the enzymes responsible for glycan biosynthesis (45). The presence and distribution of various surface glycan moieties on corneocytes were thus detected utilizing a lectin-labeling technique. As noted in Fig. S2A and B in the supplemental material, commercially available rhodamine-labeled lectins that bind specific configurations of sugar molecules were able to detect different glycan moieties present on the surface of corneocytes from the upper layers of the stratum corneum in various distribution patterns. The results indicated that glycans persist on the surfaces of corneocytes taken from the forearm, and the labeling patterns suggested the presence of different sugar moieties in specific distribution patterns.

10.1128/mBio.02908-20.5 FIG S2 Detection of rhodamine labelled lectins on the surface of corneocytes. Patterns of glycan distribution, observed by confocal microscopy at a magnification of 40 × 0.6 (A) or 63 × 1.5 (B). Lipid-rich layers of the stratum corneum were not removed in order to obtain a realistic picture of glycan localization on the surface of native corneocytes. Man, d-mannose; Glc, d-glucose; Gal, d-galactose; Fuc, l-fucose; GlcNAc, *N-*acetylgalactosamine; Sia, sialic acid (*N*-acetylneuraminic acid). Labeling of the corneocyte periphery with a beaded appearance was obtained very prominently with Ricinus communis I (RCA I) with specificity to β-d-galactose (Gal). Peripheral labeling was also obtained with concanavalin A (ConA), peanut agglutinin (PNA), soybean agglutinin (SBA), and wheat germ agglutinin (WGA), with specificities for α-d-mannose (Man) and α-d-glucose (Glc), galactose β3-linked *N*-acetylgalactosamine (Galβ3GalNAc), α>βGalNAc, and *N-*acetylglucosamine (GlcNAc), respectively. A fine, netlike distribution was observed with Dolichos biflorus agglutinin (DBA), PNA, Ulex europaeus agglutinin (UEA), and Maackia amurensis lectin (MAA/MAL), with specificities for αGalNAc, Galβ3GalNAc, α-l-fucose (Fuc) and α-(2,3)-linked sialic acid (Sia), with a very prominent signature observed using MAA/MAL I specific for Siaα2-3Galβ1-4GlcNac. Additional diffused labeling dispersed over the corneocyte surface was observed for all the above-listed lectins, except for RCA I. The labeling was weak and uniformly distributed over the corneocyte surface with Sambucus nigra lectin (SNA/EBL I), which recognizes the α-(2,6)-linked Sia (image not shown). Download FIG S2, PDF file, 0.8 MB. Copyright © 2021 Roy et al. 2021 Roy et al. https://creativecommons.org/licenses/by/4.0/ This content is distributed under the terms of the Creative Commons Attribution 4.0 International license.

### S. epidermidis binding of corneocytes after elimination of specific glycan structures by glycosidases.

**FIG 7:**
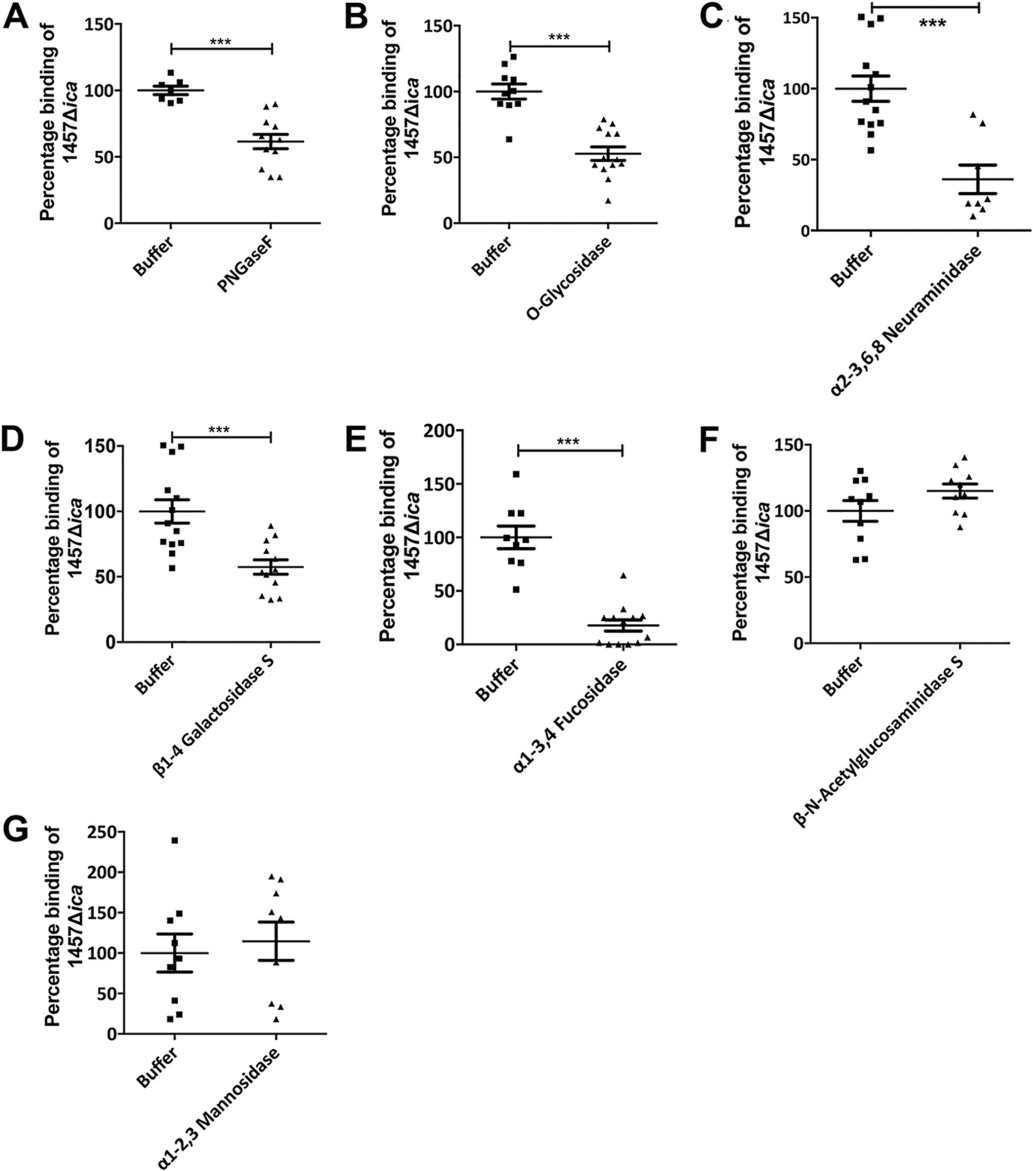
S. epidermidis corneocyte adherence following glycosidase treatment. Corneocytes were treated with specific glycosidases (A to G), and the number of adherent 1457 Δ*ica*/pCM29 bacteria after deglycosylation was normalized to the mean number of 1457 Δ*ica*/pCM29 bacteria adherent to corneocytes preincubated with buffer only (considered 100). Comparisons were performed by two-tailed unpaired Student’s *t* test with 95% confidence intervals. ***, *P* < 0.0001.

In human skin, *N*-linked glycosylation is the most common type of posttranslational modification on Asn-X-Ser/Thr motifs of epidermal proteins (46). A second major glycosylation found on the surface of keratinocytes is the covalent *O*-linkage to Ser/Thr residues of proteins (47). We reasoned that enzymatic treatment of corneocyte surfaces prior to addition of S. epidermidis with the endoglycosidases PNGase F (removes *N*-linked oligosaccharides from glycoproteins, cleaving between the innermost GlcNAc and asparagine residue) and/or *O*-glycosidase (removes core 1 and core 3 *O*-linked disaccharides from glycoproteins) would result in reduced adherence of the 1457 Δ*ica* strain to corneocytes, Indeed, enzymatic treatment with both PNGase and *O-*Glycosidase reduced adherence of the 1457 Δ*ica* strain by approximately 50%, suggesting that both of these glycan linkages expressed in the host SC are important in S. epidermidis adherence (Fig. 7A and B).

Based on these results, corneocytes were pretreated with specific exoglycosidases that catalyze hydrolysis of particular terminal sugar residues from glycoforms to assess the involvement of 5-*N*-acetylneuraminic acid (sialic acid), fucose, galactose, *N*-acetylglucosamine, and mannose terminal sugars in binding S. epidermidis. Significant reduction in the 1457 Δ*ica* strain binding was observed in α2-3,6,8 neuraminidase-, α1-3,4 fucosidase-, and β1-4 galactosidase S-pretreated corneocytes (Fig. 7C to E), confirming the importance of 5-*N*-acetylneuraminic acid, fucose, and galactose in contributing to ligand configuration, either individually or together. In contrast, pretreatment of corneocytes with β-*N*-acetylglucosaminidase S and α1-2,3 mannosidase, to remove *N*-acetylglucosamine and mannose residues, respectively, did not result in any reduction in binding of the 1457 Δ*ica* strain to corneocytes (Fig. 7F and G), suggesting that these residues either do not function in forming the ligand structure or their removal does not prevent the bacterial receptor from binding the ligand in the corneocytes. To confirm the activity of specific glycosidase treatment, corneocytes were stained with rhodamine-labeled lectins following enzymatic treatment and thus documenting reduced staining (see Fig. S3 in the supplemental material).

10.1128/mBio.02908-20.6 FIG S3 Detection of rhodamine-labeled lectins on the surface of corneocytes following treatment with glycosidases. Patterns of glycan distribution, observed by confocal microscopy at a magnification of 40 × 0.6 (A) or 63 × 1.5 (B). Lipid-rich layers of the stratum corneum were not removed. Note decreased staining of specific lectins following glycosidase treatment. Rhodamine-labeled lectins utilized are shown on the left side of the figure. Download FIG S3, TIF file, 1.5 MB. Copyright © 2021 Roy et al. 2021 Roy et al. https://creativecommons.org/licenses/by/4.0/ This content is distributed under the terms of the Creative Commons Attribution 4.0 International license.

### Genome analysis of predominant coagulase-negative staphylococci.

**FIG 8:**
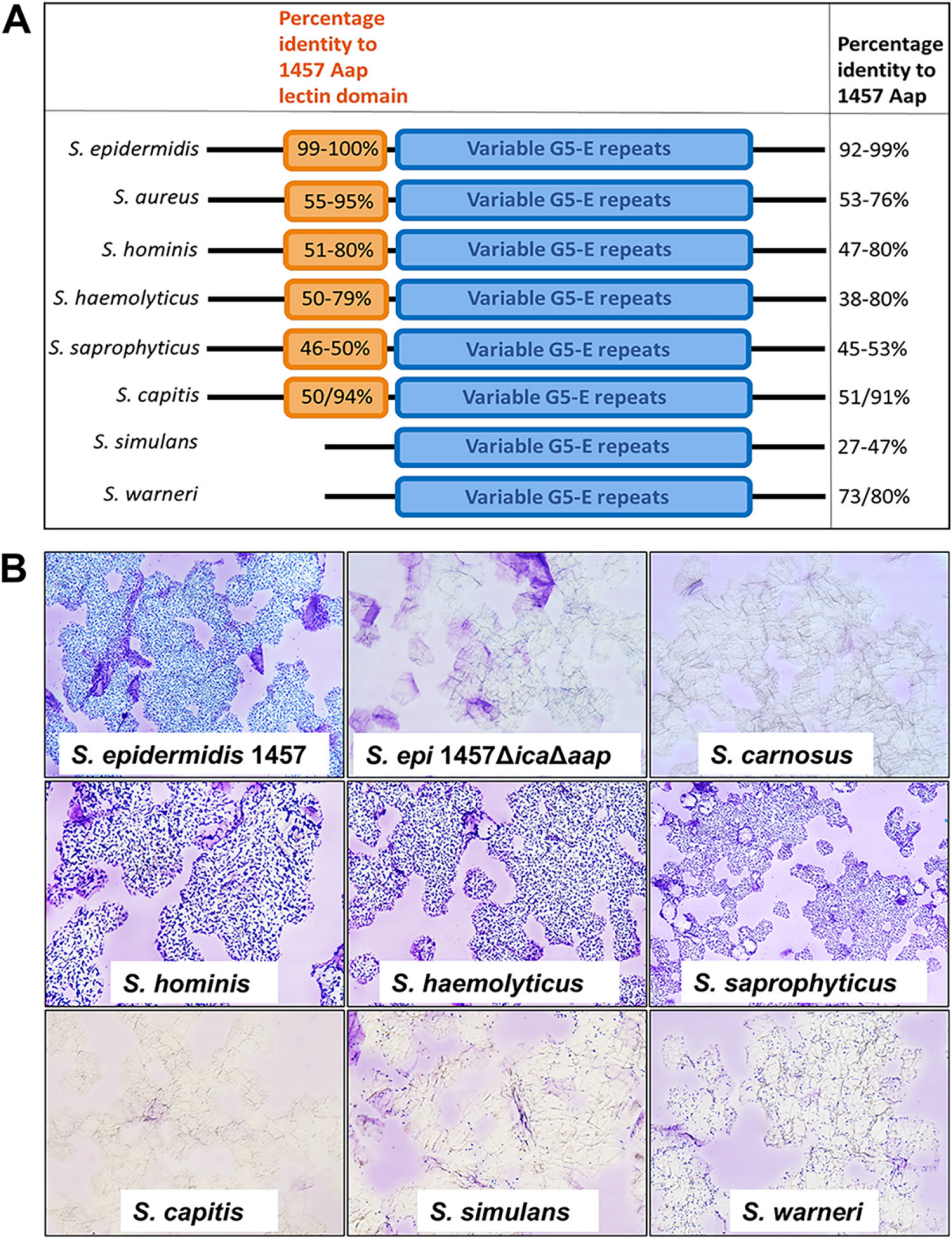
Coagulase-negative staphylococci adherence to corneocytes. (A) Representative image of genome analysis of predominant commensal coagulase-negative staphylococcal species. (B) Corneocyte binding assay visualized after crystal violet staining under light microscopy with ×40 magnification objective. The S. epidermidis 1457 Δ*ica* strain was used as a positive control, whereas 1457 Δ*ica* Δ*aap* and the adhesion-negative Staphylococcus carnosus served as negative controls.

The presence of protein sequences homologous to that of S. epidermidis 1457 Aap (GenBank accession no. ARG65518.1) in common staphylococcal skin commensals was investigated by bioinformatics analyses. The protein was considered present when a BLAST match with an alignment score of ≥200 was recorded, as shown in Table S3 in the supplemental material. Sequence similarity of the lectin domain of these homologous proteins was also compared to the 1457 Aap lectin domain to ensure elimination of Aap-like protein sequences having only G5-E domains and no lectin domain. As shown in Fig. 8A, S. hominis and S. haemolyticus strains have homologous Aap sequences with high percent identities to S. epidermidis 1457 Aap, as well as to the Aap lectin domain. Aap homologues, including the lectin domain, were also found in strains of Staphylococcus saprophyticus and S. capitis. On the other hand, Staphylococcus simulans has a protein in most strains annotated as “E domain-containing protein,” with 27 to 47% homology to 1457 Aap. However, this predicted protein consists of only G5 domains. The lectin domain is not present in any of the Aap homologous protein sequences, nor as a part of any other proteins in S. simulans. Likewise, two strains of Staphylococcus warneri were also found to encode an Aap-like protein with only G5 domains and no lectin domain. Therefore, corneocyte binding assays were performed using crystal violet staining and light microscopy (Fig. 8B). These assays documented that S. hominis, S. haemolyticus, and S. saprophyticus bound to the corneocytes similarly to the 1457 Δ*ica* strain, whereas S. capitis, S. simulans, and S. warneri exhibited either decreased (*S. capitis* and *S. simulans*) or peripheral adherence (*S. warneri*) (Fig. 8B). Overall, these data suggest that the Aap orthologues may function to provide the basis for staphylococcal colonization of the skin, with the L-type lectin domain operating as receptors for specific ligands on the surface of corneocytes.

10.1128/mBio.02908-20.3 TABLE S3 Sequence homology of S. epidermidis 1457 Aap and its lectin domain with other staphylococcal skin commensals. Download Table S3, DOCX file, 0.02 MB. Copyright © 2021 Roy et al. 2021 Roy et al. https://creativecommons.org/licenses/by/4.0/ This content is distributed under the terms of the Creative Commons Attribution 4.0 International license.

### Corneocyte binding of S. aureus.

**FIG 9:**
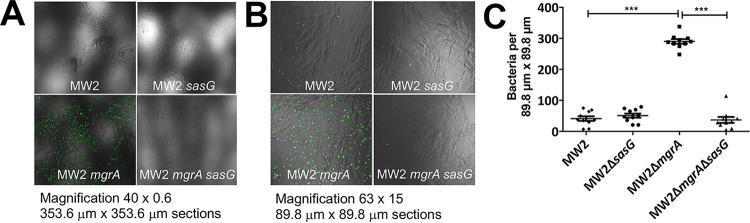
Derepression of SasG in the MW2 Δ*mgrA* strain significantly increases adherence of S. aureus to corneocytes. (A) Corneocyte binding assay performed with S. aureus MW2/pCM29 and mutants, visualized by confocal microscopy at 40 × 0.6 magnification and (B) 63 × 1.5 magnification. (C) Mean number (with SEM) of MW2/pCM29 and mutants adhered to corneocytes (per 89.8- μm × 89.8-μm sections) after corneocyte binding assay. Comparisons between individual mutants were performed by two-tailed unpaired Student’s *t* test with 95% confidence intervals. ***, *P* < 0.0001.

Despite possessing the Aap orthologue SasG, S. aureus is not a normal component of the normal human skin microbiota. Indeed, many S. aureus strains, including JE2, have premature stop codons within *sasG*, which presumably result in a nonfunctional protein. Therefore, we chose to work with S. aureus MW2, which has been previously shown to express a functional SasG (48). As shown in Fig. 9, S. aureus MW2 and MW2 Δ*sasG* strains displayed negligible adherence to corneocytes. However, it has been previously demonstrated that the transcription of *sasG*, as well as that of seven other cell wall-associated proteins, is repressed by the global regulator MgrA, which in turn is regulated by the ArlRS two-component regulatory system (48). Thus, the contribution of MgrA was assessed in the corneocyte adherence assay. Indeed, the binding of MW2 increased significantly in the MW2 Δ*mgrA* mutant. This increase in adherence was confirmed to be SasG dependent, as the phenotype of the MW2 Δ*mgrA* Δ*sasG* mutant phenocopied that of MW2, suggesting that derepression of SasG in the absence of MgrA can significantly increase the binding of S. aureus to corneocytes. Thus, these data confirm that when SasG is expressed, it is able to mediate significant binding of S. aureus to corneocytes, similarly to S. epidermidis, reiterating the importance of Aap and its orthologues in the adherence of staphylococci to healthy skin.

## DISCUSSION

There is emerging evidence that S. epidermidis and other commensal staphylococci ubiquitously present on cutaneous surfaces inhibit the colonization of harmful pathogenic microorganisms (9–14, 49). Although pathogen inhibition clearly involves production of inhibitory compounds by CoNS and other commensals, we surmise that adherence to corneocytes, and potentially intercellular aggregation, is a prerequisite for secretion of these compounds. Furthermore, S. epidermidis and other commensals may have evolved to adhere to specific ligands, thus successfully outcompeting S. aureus. Therefore, we propose that determining how S. epidermidis adheres to corneocytes is a missing component of understanding the pathogenesis of S. aureus skin and soft tissue infections. Indeed, understanding these interactions may reveal how S. aureus evolved to counteract inhibition by S. epidermidis and other CoNS.

This study demonstrated that Aap is a critical adhesin that mediates adherence of S. epidermidis 1457 to corneocytes. Our experiments confirmed that the Aap-mediated adherence of 1457 to corneocytes occurs primarily via interactions of the A domain with corneocytes. The conserved globular lectin-like subdomain was found to mediate adherence to surface ligands in the corneocytes and confer the A domain with optimum binding capacity. Inhibition of bacterial adherence using anti-Aap antiserum specific for either the A or B domains or the use of recombinant domain proteins provided results documenting the importance of the complete A domain and the lectin subdomain in the binding process. However, failure to completely prevent bacterial adherence even after blocking with a high concentration of the rA_rep+lec_ domain (10 μM) (Fig. 3C), in contrast to the nearly complete blocking of adherence via anti-AapA domain antiserum (Fig. 2A), suggested loss of structural integrity or reduced binding affinity in the recombinant soluble form of the A_rep+lec_ domain. Interestingly, 50% blocking required 2.48 μM rA_rep+lec_ domain compared to 7.54 μM rA_lectin_ domain. Therefore, a lower concentration of the rA_rep+lec_ domain than the rA_lectin_ domain is required to bind half the available ligands present on corneocytes, indicating that the rA_rep+lec_ domain has higher blocking efficiency than the rA_lectin_ domain. Therefore, we speculate that the complete rA_rep+lec_ domain might possess a higher affinity for the ligand on the corneocyte than rA_lectin_ domain alone and hypothesize that the 16-amino-acid repeat region present N-terminally to the lectin domain provides conformational precision for the lectin domain to bind the ligand in the stratum corneum of the skin. Alternatively, since the Aap lectin domain is predicted to possess homodimer and homotetramer interaction sites for oligomeric interactions (predicted by the NCBI Conserved Domains Database), multiple copies of Aap lectin domains expressed together in S. epidermidis might show multivalent interactions with corneocyte ligands with higher binding avidity than that of the monovalent recombinant forms. Corneocyte binding assays performed using isogenic mutants expressing truncated variants of Aap confirmed that while the lectin subdomain binds to corneocytes, the N-terminal A-repeat region also functions to provide the lectin domain with the maximum binding ability, and the B domain functions, presumably, to extend the A domain, facilitating interaction with the corneocyte ligand.

Many studies have suggested that infection or colonization by bacteria is generally initiated by the specific recognition of host epithelial surfaces by adhesins and lectins. Glycans are present on all cell types and have specific biological functions in cell-cell recognition and cell-matrix interactions within the host (50). Pathogens have evolved to exploit these cell surface glycans by recognizing specific sequences of glycans as targets to bind host cells. Adhesins expressed on the surface of these microorganisms are often equipped with lectin domains that mediate binding to such glycans presented on proteins or lipids on the host surface (50). We hypothesize that the lectin-like domain of Aap binds glycans present on the surface of the corneocytes. The use of glycosidases suggested that the terminal glycans expressed in the host stratum corneum serve as binding partners for the Aap-mediated colonization of S. epidermidis on the corneocyte surface. Indeed, antibodies against the highly expressed corneocyte proteins loricrin and cytokeratin-10 had no effect on S. epidermidis adherence (see Fig. S4 in the supplemental material). Given the terminal location of the negatively charged sialic acid in most human glycoforms and their widespread distribution, sialic acids are often the targets for binding by a large number of pathogenic organisms and their toxins (51). Hence, we hypothesize that Neu5Ac might present an important structure for S. epidermidis ligand in corneocytes. Galactose, GlcNAc, and fucose also form terminal “decorating” sugars, and thus we speculate the involvement of these sugars in S. epidermidis adherence. It should also be noted that the complete loss of S. epidermidis adherence to corneocytes was not observed following glycosidase treatment (Fig. 7). These results may suggest an incomplete removal of glycan moieties by the endoglycosidases, probably due to the difficulty in accessing the interior target in the presence of lipid-rich layers on the corneocyte sheets. Alternatively, it might suggest that glycolipids, in addition to glycoproteins, have a function in S. epidermidis adherence. Taken together, the results generated by this study demonstrate that the adherence of S. epidermidis to corneocytes is dependent on terminal sugar residues, with sialic acid, fucose, and galactose being the most promising binding ligands (Fig. 7).

10.1128/mBio.02908-20.7 FIG S4 The major corneocyte proteins do not have a function in adherence of S. epidermidis. (A) Cytokeratin-10 and (B) loricrin are the major corneocyte proteins, observed by confocal microscopy at a magnification of 40 × 0.6. (C) Preincubation of corneocytes with 10 μg/ml antibody against cytokeratin-10 and loricrin for 1 h at 37°C prior to incubation with bacteria resulted in similar bacterial counts as control with 1% bovine serum albumin (BSA), suggesting that these proteins do not play a role in binding to S. epidermidis. Download FIG S4, JPG file, 0.5 MB. Copyright © 2021 Roy et al. 2021 Roy et al. https://creativecommons.org/licenses/by/4.0/ This content is distributed under the terms of the Creative Commons Attribution 4.0 International license.

**FIG 10:**
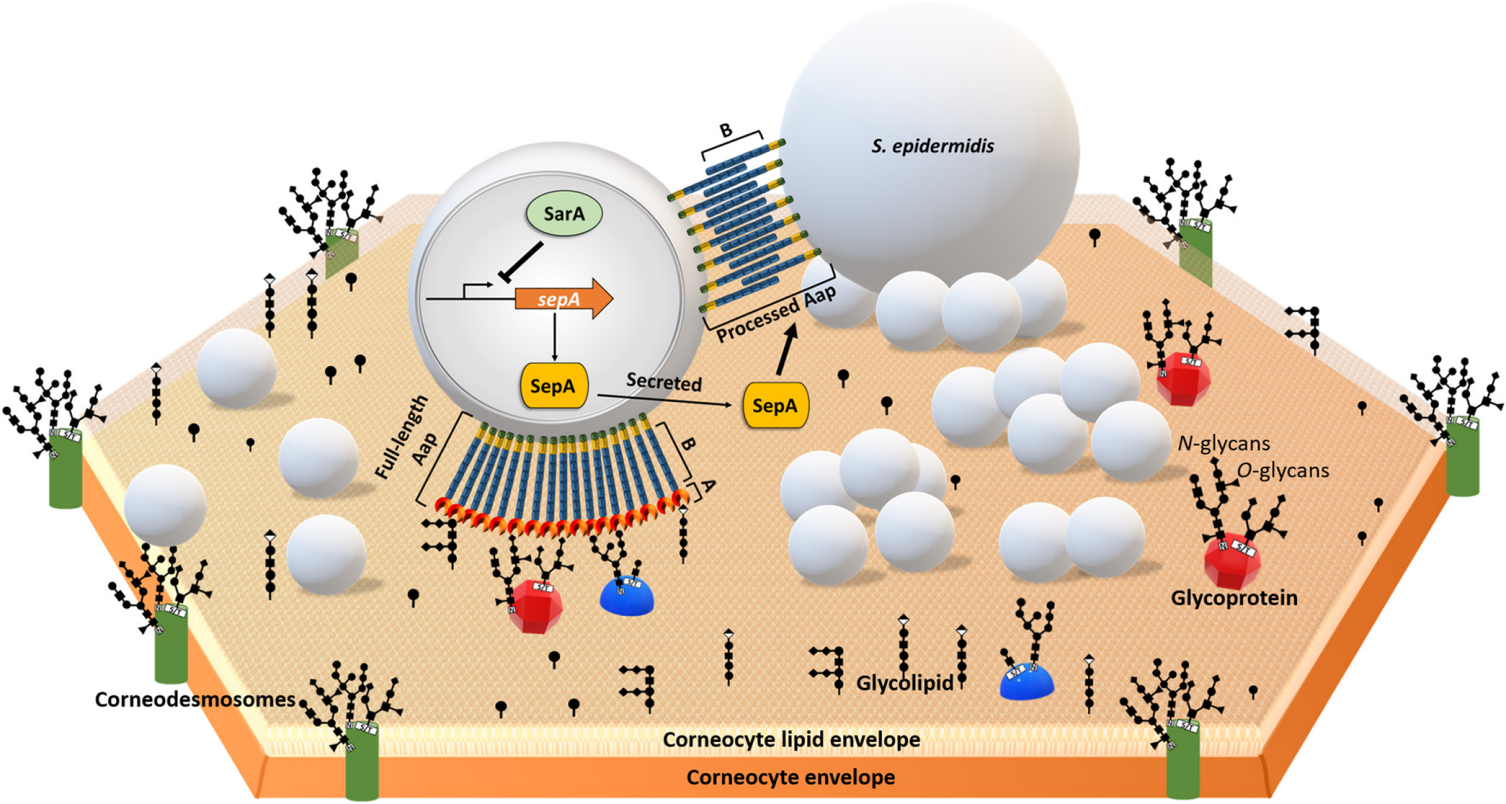
S. epidermidis adherence to corneocytes. A hypothetical model of S. epidermidis binding to glycans linked to proteins or lipids that are expressed on the surface of corneocytes. It is proposed that S. epidermidis expressing the full-length Aap on its cell wall adheres to corneocytes from the uppermost layers of the skin epidermis via the A domain. Aap A domain-mediated binding is dependent upon the lectin subdomain but requires the A-repeat domain and the B domain for maximum ligand-receptor interaction. Aap may bind to a specific glycan molecule or bind to multiple glycan molecules with different affinities. Furthermore, SepA, which is regulated by SarA, processes Aap, facilitating B domain-mediated interactions and subsequent cellular aggregation on the corneocyte surface.

Aap is a multifunctional protein that contributes to both primary attachment and subsequent biofilm accumulation (32, 33, 35). These well-defined functions are mediated by its structurally distinct A and B domains (29, 36, 37). Studies show that expression of full-length Aap mediates attachment of S. epidermidis to uncoated abiotic surfaces, presumably through hydrophobic interactions via the A domain (32, 33). Additionally, Schaeffer et al. found that S. epidermidis adherence to a rat jugular catheter was dependent upon Aap (33). It is unclear, however, if the same glycan expressed on corneocytes is also coated on catheters or whether Aap binds multiple glycan ligands. Additionally, Aap is subjected to proteolytic processing by the SepA metalloprotease (or by host-derived proteases), which results in distinct isoforms of the expressed protein (33–35). Based on Western blot analysis (34), we predict that about half of the cell wall-associated Aap molecules are processed by SepA. Thereafter, processed Aap can participate in Zn^2+^-dependent intercellular adhesion via the B domains to facilitate cellular aggregation and/or biofilm accumulation in S. epidermidis. Moreover, SepA-mediated proteolysis of Aap within the A domain (at Leu 335) and at the junction between the A and B domains (at Leu 601) is under the regulation of the global transcriptional regulator, SarA, thus allowing S. epidermidis to fine-tune its biofilm-inducing properties in response to environmental cues (34). However, it is unclear if Aap processing via SepA has a specific function with regard to S. epidermidis adherence to corneocytes. We propose that, following A lectin-mediated adherence to the glycan, processed Aap facilitates additional cellular aggregation allowing for increased colonization of the skin surface (Fig. 10). Indeed, Gonzalez and colleagues recently found via scanning electron microscopy that bacteria colonizing skin were observed as aggregates of cells, suggesting that subsequent B domain-mediated aggregation may be important for skin surface colonization (52).

Commensal staphylococcal species possessing homologous Aap adhesins with a functional lectin-like domain may also function to bind corneocyte glycan ligands, similarly to S. epidermidis. We were able to confirm this in S. aureus MW2 strain expressing SasG, an orthologue of Aap. Though L-type lectins exhibit both sequence and structural similarity to one another, their carbohydrate binding specificities differ widely depending on the residues of the adjoining sequence of the carbohydrate binding site. Thus, it is possible that each CoNS species exhibits specificity for distinct sugar residues or moieties, which might explain the observed binding pattern differences between different strains. Alternatively, SasG and Aap (or other staphylococcal Aap orthologues) may bind similar glycan moieties, and competition for these binding sites may exist. This may suggest that S. aureus is selected to repress SasG via MgrA to avoid competition with S. epidermidis and/or other coagulase-negative staphylococcal species. Indeed, many strain backgrounds, including USA300, the most prominent S. aureus lineage causing infections in the United States, encode a truncated and presumably nonfunctional SasG protein, suggesting that certain populations of S. aureus may use alternative strategies to facilitate corneocyte adherence (48, 53).

Conventionally, glycan recognition by proteins is of low affinity compared to protein-protein interactions, which typically have high binding affinities. However, since adhesins and ligands often cluster in the plane of the membrane, the resulting combinatorial avidity can be high (50, 54). It is also possible that S. epidermidis has adhesins that promote glycan-independent mechanisms of interaction with the host corneocyte proteins, in a similar manner as SdrF binding to cytokeratins (39). Nevertheless, glycan-dependent binding mechanisms often function to play a role in the first step of adhesion, prior to invasion during infections. It can also be advantageous in harsh environmental conditions where opportunities for contact and adherence may be transient and difficult (53, 54).

In conclusion, S. aureus is the most significant cause worldwide of skin and skin structure infections. However, S. aureus is infrequently isolated from the skin from healthy subjects. This suggests that S. epidermidis and other bacteria colonizing the skin are highly effective at inhibiting colonization of this pathogen. We found that S. epidermidis binds to healthy corneocytes using Aap and that other coagulase negative staphylococci also contain an Aap orthologue, suggesting a potential common mechanism for corneocyte adherence. These data may suggest that S. epidermidis has the ability to outcompete S. aureus, potentially via adherence to carbohydrate molecules decorating corneocytes. Further studies will focus on competition between these two species, determine whether SasG and Aap bind similar ligands and, potentially, determine if S. aureus uses other adhesins to bind healthy or diseased skin.

## MATERIALS AND METHODS

### Bacterial strains and medium.

Bacterial strains and plasmids used in these studies are described in Table S1 in the supplemental material. S. epidermidis strains were grown in tryptic soy broth (TSB) or agar (Difco). Escherichia coli DH5α, used for cloning purposes, and Escherichia coli BL21(DE3), used for overexpressing recombinant proteins, was grown in lysogeny broth or agar (LB; Difco). Antibiotics and concentrations for cloning and selection purposes were as follows: ampicillin (50 μg/ml), kanamycin (100 μg/ml), tetracycline (5 μg/ml), erythromycin (10 μg/ml), chloramphenicol (10 μg/ml), and trimethoprim (10 μg/ml). Cultures were grown aerobically (1:10 medium-to-flask ratio, 250 rpm) at 37°C or at 30°C (for temperature-sensitive strains).

10.1128/mBio.02908-20.1 TABLE S1 Bacterial strains, plasmids, and bacteriophages used in this study. Download Table S1, DOCX file, 0.04 MB. Copyright © 2021 Roy et al. 2021 Roy et al. https://creativecommons.org/licenses/by/4.0/ This content is distributed under the terms of the Creative Commons Attribution 4.0 International license.

### Construction of recombinant His_6_-tagged fusion proteins.

First, a 1,602-bp fragment containing the A domain sequence of *aap* from S. epidermidis 1457 (55) was amplified using primers r-*aap*Adom F and r-*aap*Adom R. Similarly, 915 bp of the A-repeat region of *aap* was amplified using the primers r-*aap*Adom F and r-*aap*Arep R, and 681 bp of the L-type lectin domain of *aap* was amplified using the primers r-*aap*Lec F and r-*aap*Adom R. PCR was carried out using chromosomal DNA from S. epidermidis strain 1457 as the template and Q5 high-fidelity DNA polymerase (New England Biolabs [NEB], Ipswich, MA) according to the manufacturer’s instructions. The primers used are listed in Table S2 in the supplemental material. The resulting amplicons were cloned into pET28a using the NdeI and XhoI restriction sites, creating plasmids pNF319, pNF326, and pNF318 for N-terminal His_6_-tagged rA_rep+lec_ domain, rA_repeat_ subdomain, and rA_lectin_ subdomain, respectively, in chemically competent E. coli DH5α cells. The primers T7Promoter F and T7Terminator R were used for confirming the inserts by PCR. Plasmid DNA was isolated from E. coli DH5α using the Wizard Plus SV Minipreps DNA purification system (Promega, Madison, WI) according to the manufacturer’s recommendations. The plasmids were transformed into chemically competent E. coli BL21(DE3) cells for overexpression of the recombinant proteins.

10.1128/mBio.02908-20.2 TABLE S2 Primers used in this study. Download Table S2, DOCX file, 0.02 MB. Copyright © 2021 Roy et al. 2021 Roy et al. https://creativecommons.org/licenses/by/4.0/ This content is distributed under the terms of the Creative Commons Attribution 4.0 International license.

### Expression and purification of recombinant proteins.

For recombinant expression of the A_rep+lec_ domain and subdomains A_repeat_ and A_lectin_, BL21(DE3)/pNF319, BL21(DE3)/pNF326, and BL21(DE3)/pNF318 were induced using autoinduction medium (adapted from Studier [56]) for 18 h at 37°C under aerobic conditions. Induced cultures were pelleted and resuspended for lysis in 6 ml of lysis buffer containing BugBuster protein extraction reagent (EMD Millipore, Billerica, MA), EDTA-free cOmplete protease inhibitor cocktail (Roche, Mannheim, Germany), and benzonase nuclease (EMD Millipore), for 20 min at room temperature under gentle shaking conditions. Samples were then centrifuged at 4°C and supernatants incubated with 1-ml resins charged with divalent cobalt provided in HisPur cobalt spin columns (Pierce Biotechnology, Rockford, IL) overnight on an end-over-end mixer at 4°C for affinity purification of the recombinant proteins. Columns were then washed and eluted following the manufacturer’s instructions. Affinity-purified proteins were concentrated at 20°C using a Vivaspin 10,000-molecular weight cutoff (MWCO) column (Sartorius AG; Göttingen, Germany) and stored in phosphate-buffered saline (PBS) with 30% glycerol at −20°C. SDS-PAGE and subsequent Coomassie staining tested the integrity of the respective protein preparations.

### Construction of *srtA* and *sdrF* allelic replacement knockout mutants in strain 1457.

To construct the *sdrF* allelic replacement plasmid, PCR was performed using chromosomal DNA from S. epidermidis 1457 as the template and Phusion high-fidelity DNA polymerase (NEB) according to the manufacturer’s instructions. The primers used are listed in Table S2. A 1,001-bp fragment, containing the 3′ region of *sdrF* and downstream sequence, was amplified using the primers *sdrF*DN F and *sdrF*DN R. The fragment was inserted into the XmaI and SalI sites of the shuttle vector pJB38 (57), creating plasmid pNF360. A 1,001-bp fragment, containing the 5′ region of *sdrF* and upstream sequence, was amplified using the primers *sdrF*UP F and *sdrF*UP R. The fragment was inserted into pNF360 between the SacI and XmaI sites, creating pNF361. A 1,260-bp fragment containing the *ermC* cassette was amplified from pROJ6448 (58) with XmaI ends using the primers *ermC-*XmaI F and *ermC-*XmaI R. The fragment was inserted in the XmaI site of pNF361, creating the *sdrF* allelic replacement plasmid pNF362. The inserts were confirmed by restriction analysis and PCR with the primers pJB38seq F and pJB38seq R. Subsequently, pNF362 was electroporated into electrocompetent PS187 cells (59) (0.1-cm cuvette, 100 Ω, 25 μF, and 2.3 kV) (60), followed by transduction into the S. epidermidis 1457 *icaADBC*::*dhfr aap*::*tetM* strain (33) with bacteriophage Φ187 (59), using a previously described protocol (61). Allelic exchange was performed thereafter to generate single and double recombinants, as previously described (62). Single recombinants were selected for resistance to trimethoprim, tetracycline, erythromycin, and chloramphenicol, while the allelic replacement mutant generated from double recombination was selected based on loss of resistance to chloramphenicol alone (plasmid marker). Following allelic replacement, the *sdrF* mutant was backcrossed into the 1457 *icaADBC*::*dhfr* (63) and 1457 *icaADBC*::*dhfr aap*::*tetM* strains using the transducing phage Φ71 (64), to generate the 1457 *icaADBC*::*dhfr sdrF*::*ermC* and 1457 *icaADBC*::*dhfr aap*::*tetM sdrF*::*ermC* mutants, respectively. The allelic replacement mutants were confirmed by PCR with the primers *sdrF*seq F and *sdrF*seq R and pulsed-field gel electrophoresis (PFGE) as previously described (65).

To construct the *srtA* allelic replacement plasmid, PCR was performed using chromosomal DNA from S. epidermidis 1457 as the template and *Taq* DNA polymerase. The primers used are listed in Table S2. A 2,074-bp fragment containing the *srtA* gene and its upstream and downstream sequence was amplified with primers *srtA*UP F and *srtA*DN R and cloned into pCR2.1 by TA cloning, creating plasmid pNF79. The insert flanked by EcoRI sites was excised from pNF79 and cloned into the EcoRI site of pUC19, creating plasmid pNF82. A 2,383-bp *tetM* cassette was amplified from pNF105 with ClaI ends using the primers *tetM-*ClaI F and *tetM-*ClaI R and inserted into the ClaI site in the middle of the *srtA* gene of pNF82, creating plasmid pNF107. Finally, pROJ6448, containing a temperature-sensitive origin of replication and conferring erythromycin resistance, was linearized with PstI and ligated into the PstI site of pNF107, generating the *srtA* allelic replacement plasmid pNF112. All of the above-described cloning were done in E. coli DH5α cells. Subsequently, pNF112 was electroporated into electrocompetent PS187 cells (0.1-cm cuvette, 100 Ω, 25 μF, and 2.3 kV) and then transduced into S. epidermidis 1457 using bacteriophage Φ187. Allelic exchange was performed thereafter to generate single and double recombinants. Single recombinants were selected for resistance to tetracycline and erythromycin. The allelic replacement mutant generated from double recombination was able to grow on tryptic soy agar with tetracycline (TSA+Tet) but not on TSA+Erm. Following allelic replacement, the *srtA* mutant was backcrossed into the 1457 and 1457 *icaADBC*::*dhfr* strains, using the transducing phage Φ71, to generate the 1457 *srtA*::*tetM* and 1457 *icaADBC*::*dhfr srtA*::*tetM* mutants, respectively. The allelic replacement mutants were confirmed by PCR and PFGE.

### Construction of *aap* allelic replacement knock-in mutants expressing truncated variants of Aap in the 1457 Δ*ica* strain.

Isogenic mutants expressing truncated variants of Aap from i to vi were generated by insertion of plasmid constructs containing specific combinations of *aap* domains (markerless) into the 1457 *icaADBC*::*dhfr aap*::*tetM* construct by allelic replacement of Δ*aap*::*tetM*. All of the allelic exchange constructs contained the upstream and downstream region of *aap* for upstream and downstream homologous recombination, respectively. Allelic exchange constructs for the following mutants were made as described below. PCR amplifications were carried out using chromosomal DNA from S. epidermidis 1457 as the template and one of the following polymerases: Q5 high-fidelity DNA polymerase (NEB), Pfurther long-range DNA polymerase (Monserate Biotechnology Group, San Diego, CA), or Phusion high-fidelity DNA polymerase (NEB). The primers used are listed in Table S2.

### (i) The 1457 Δ*ica aap*::*A*-(*rep*+*lec*) construct.

For the upstream insert, a 2,609-bp fragment comprising the *aap* upstream region (UP), the signal sequence (SS), and the complete A-domain sequence of *aap* was amplified with primers *aap*UP F1 and *aap*Adom R. The amplified fragment was inserted in between the EcoRI and XmaI restriction site of the shuttle vector pJB38, to create pNF344. For the downstream insert, a 1,369-bp fragment comprising the PGR region, the LPDTG Gram-positive anchor domain, and the downstream region (DN) of *aap* was amplified with the primers *aap*PGR F and *aap*DN R. This fragment was inserted into the XmaI and SalI site of pNF344 to generate pNF348 [*aap*A-(*rep*+*lec*) allelic exchange vector]. pNF348 was used to create a B domain-lacking 1457 Δ*ica aap*::*A-*(*rep+lec*) mutant for expressing a truncated variant of Aap with only the A domain attached to the PGR stalk and the LPDTG Gram-positive anchor.

### (ii) The 1457 Δ*ica aap*::*B* construct.

For the upstream insert, a 1,001-bp fragment comprising the *aap* UP and SS of *aap* was amplified with primers *aap*UP F and *aap*SS R. The fragment was cloned into the EcoRI and XmaI site of pJB38 to create pNF334. For the downstream insert, a 3,940-bp fragment containing the B domain, PGR region, and LPDTG Gram-positive anchor domain of *aap*, along with the *aap* DN, was amplified with *aap*Bdom F and *aap*DN R. This fragment was cloned into the XmaI and SalI site of pNF334, generating pNF343 (*aap*B allelic exchange vector). pNF343 was used to create an A domain-lacking 1457 Δ*ica aap*::*B* mutant for expressing a truncated variant of Aap with only the B domain attached to the PGR stalk and LPDTG Gram-positive anchor.

### (iii) The 1457 Δ*ica aap*::*A-rep* construct.

For the upstream insert, a 1,916-bp fragment comprising the *aap* UP, the SS, and the A-repeat region of *aap* was amplified with the primers *aap*UP F1 and *aap*Arep R. The fragment was cloned into the EcoRI and XmaI site of pJB38 to create pNF346. For the downstream insert, the 1,369-bp [*aap*(PGR+LPDTG)+DN] fragment amplified previously with the primers *aap*PGR F and *aap*DN R, was cloned into the XmaI and SalI site of pNF346 to generate pNF347 (*aap*A-*rep* allelic exchange vector). pNF347 was used to create the 1457 Δ*ica aap*::*A-rep* mutant for expressing a truncated variant of Aap with only the A-repeat region attached to the PGR stalk and LPDTG Gram-positive anchor, devoid of the A-lectin subdomain and the B domain.

### (iv) The 1457 Δ*ica aap*::*A-lec* construct.

The 1,369-bp insert [*aap*(PGR+LPDTG)+DN] amplified above with primers *aap*PGR F and *aap*DN R, was cloned into the XmaI and SalI site of the previously generated pNF334, creating pNF345. Next, 687 bp of the lectin domain sequence of *aap* was amplified with *aap*Alec F and *aap*Adom R and cloned into the XmaI site of pNF345. The directionality of the *aap*A-lectin insert was checked with aapAlecOut and *aap*UP F. The resulting generated construct was named pNF350 (*aap*A*-lec* allelic exchange vector). pNF350 was used to create the 1457 Δ *ica aap*::*A-lec* mutant for expressing a truncated variant of Aap with only the A-lectin subdomain attached to the PGR stalk and LPDTG Gram-positive anchor, devoid of the A-repeat region and the B domain.

### (v) The 1457 Δ*ica aap*::*A-rep+B* construct.

The 3,940-bp fragment containing [*aap*(B+PGR+ LPDTG)+DN], amplified previously with *aap*Bdom F and *aap*DN R, was cloned into the XmaI and SalI site of the previously generated pNF346, creating pNF349 (*aap*A-*rep*+*B* allelic exchange vector). pNF349 was used to create an A-lectin subdomain-lacking 1457 Δ*ica aap*::*A-rep+B* mutant for expressing a truncated variant of Aap with the A-repeat region attached to the B domain, PGR stalk, and LPDTG Gram-positive anchor.

### (vi) The 1457 Δ*ica aap*::*A-lec+B* construct.

A 4,648-bp fragment comprising the A-lectin subdomain, B domain, PGR region, and LPDTG Gram-positive anchor of *aap*, along with *aap* DN, was amplified with primers *aap*Alec F and *aap*DN R. This fragment was cloned into the XmaI and SalI site of the previously made pNF334, generating pNF342 (*aap*A-*lec*+*B* allelic exchange vector). pNF342 was used to create an A-repeat region-lacking 1457 Δ*ica aap*::*A-lec+B* mutant for expressing a truncated variant of Aap with the A-lectin subdomain attached to the B domain, PGR stalk, and LPDTG Gram-positive anchor.

All of the above allelic exchange plasmids were constructed in E. coli DH5α cells. The inserts were confirmed by restriction analysis and PCR with the primers pJB38seq F and pJB38seq R. These plasmids were electroporated into electrocompetent PS187 cells (0.1-cm cuvette, 100 Ω, 25 μF, and 2.3 kV) and then transduced into the S. epidermidis 1457 *icaADBC*::*dhfr aap*::*tetM* mutant using bacteriophage Φ187. Allelic exchange was performed thereafter to generate single and double recombinants. Single recombinants were selected for resistance to trimethoprim and tetracycline and chloramphenicol, while the knock-in mutants generated from double recombination were selected based on loss of resistance to tetracycline and chloramphenicol. The mutants thus generated (i to vi) were confirmed by PCR with the primers *aap*UPseq F and *aap*DNseq R, PFGE, and immunoblotting with antibodies to Aap.

### (vii) The 1457 Δ*ica aap*::*A*-(*rep+lec*)*+B* construct.

pNF344 containing the insert [UP+*aap*(SS+*A*-(*rep*+*lec*))] was used as the allelic exchange vector for introducing the A-repeat region back into the 1457 Δ*ica aap*::*A-lec+B* strain for regenerating the wild-type *aap* genetically. For this purpose, pNF344 was electroporated into electrocompetent PS187 and transduced into the 1457 Δ*ica aap*::*A-lec+B* mutant using bacteriophage Φ187. Allelic exchange was performed thereafter to generate single recombinants that were resistant to trimethoprim and chloramphenicol, while double recombinants were selected based on loss of chloramphenicol resistance on the plasmid. The knock-in mutants were selected from double recombinants based on PCR confirmation with the primers *aap*AlecOut and *aap*UP F. Mutant construction was also verified by PFGE and immunoblotting with Aap antibodies.

### Preparation of cell wall proteins.

Fresh TSB (12.5 ml in a 125-ml flask) was inoculated at an OD_600_ of 0.02, and the cultures were grown to the exponential phase at 37°C with shaking at 250 rpm until an OD_600_ of ∼0.7 was reached. Cells were collected by centrifugation at 5,000 rpm for 10 min at 4°C, and the pellet was washed three times with 1 ml 1× PBS (Dulbecco’s PBS, no calcium, no magnesium). To isolate cell walls, cell pellets were resuspended in 200 μl lysis buffer (50 mM Tris-HCl [pH 7.5], 20 mM MgCl_2_, 30% [wt/vol] raffinose, 1 mM EDTA, 20 U DNase I [Roche], 4 mM phenylmethylsulfonyl fluoride, 1 mM *N*-ethylmaleimide, 25 mM aminocaproic acid, lysostaphin [100 mg/ml; AMBI Products, Lawrence, NY], and lysozyme [100 mg/ml]) and incubated at 37°C with shaking for 30 min. Cell walls were separated from protoplasts by centrifugation at 6,000 × *g* for 20 min. The supernatant (cell wall fraction) was removed, and samples were stored at −20°C.

### Western blot analysis.

The concentration of protein samples was determined using a NanoDrop 2000 spectrophotometer (Thermo Scientific, Waltham, MA) or with bicinchoninic acid (BCA) assay (Pierce Biotechnology). Samples were adjusted for equal protein amount (35 μg total protein for cell wall preparations; 50 ng for purified proteins), mixed with 4× SDS sample loading buffer, denatured at 95°C for 10 min, and then loaded on a 7 to 10% SDS-PAGE gel. Current was applied at 60 V for 30 min and at 150 V until the dye front reached the bottom of the gel. Proteins were transferred onto an activated 0.45-μm polyvinylidene difluoride (PVDF) membrane in cold 1× transfer buffer at 100 V for 2 h.

Membranes were blocked with 5% nonfat milk in Tris-buffered saline with 0.1% Tween (TBS-T). Aap A- or B-domain antiserum (23) was diluted 1:100,000 in 5% nonfat milk. Alkaline phosphatase (AP)-conjugated mouse anti-rabbit IgG (Jackson ImmunoResearch, West Grove, PA) was diluted 1:25,000. Blots were developed with enhanced chemifluorescence (ECF) substrate (GE Healthcare Life Sciences; Piscataway, NJ) and visualized on a Typhoon FLA 7000 laser scanner (GE Healthcare Life Sciences).

### Collection of corneocytes.

Sheets of corneocytes were collected by applying adhesive discs (D-Squame skin indicator D232; CuDerm Corp., Dallas, TX) with a 12.7-mm-diameter sensing area, on the inner forearm of healthy human volunteers with no history or physical signs of dermatological disease. Corneocytes were collected from the selected area after removal of the topmost epidermal layer using a regular adhesive tape and subsequent topical application of isopropyl alcohol with prep pads for sterilization. The outermost layers of the stratum corneum of the forearm were chosen for our experiments, as mature and rigid cornified envelopes were detected in the upper layers, in contrast to immature and fragile cornified envelopes from the deeper layers of stratum corneum (66).

### Labeling of corneocytes.

The following rhodamine-labeled lectins were used at a final concentration of 20 μg/ml in PBS to label corneocytes: concanavalin A (ConA) recognizes α-d-mannose (Man) and α-d-glucose (Glc), Dolichos biflorus (DBA) and soybean agglutinin (SBA) recognize *N*-acetylgalactosamine (GalNAc), peanut agglutinin (PNA) recognizes galactose β(1,3)-linked *N*-acetylgalactosamine (Galβ3GalNAc), Ricinus communis I (RCA 1) recognizes β-d-galactose (Gal), Ulex europaeus I agglutinin (UEA I) recognizes α-l-fucose (Fuc), and wheat germ agglutinin (WGA) recognizes *N*-acetylglucosamine (GlcNAc). The above-listed lectins were purchased from Vector Laboratories (Burlingame, CA). Sambucus nigra lectin (SNA/EBL I) that recognizes α-(2,6)-linked sialic acid (Sia), Maackia amurensis lectin (MAA/MAL I) that recognizes α-(2,3)-linked sialic acid (Siaα2-3Galβ1-4GlcNAc), and Maackia amurensis lectin (MAA/MAL II) that recognizes Siaα2-3Galβ1-3GalNAc were purchased from GlycoMatrix (Dublin, Ohio). Corneocyte discs, with or without deglycosylation treatment of the corneocyte surfaces (see below), were incubated for 1 h, followed by removal of the stain and washing three times in PBS, prior to confocal imaging.

For visualization of cytokeratin-10 and loricrin labeling on the corneocytes, corneocyte slides were incubated with 10 μg/ml rabbit polyclonal anti-cytokeratin-10 IgG (Abcam, Cambridge, MA) or rabbit polyclonal anti-loricrin IgG (Abcam) primary antibodies for 1 h. This was followed by incubation with Alexa Fluor 555-conjugated goat anti-rabbit IgG (Thermo Scientific) secondary antibody and subsequent confocal microscopy imaging.

### Optimization of the corneocyte binding assay.

The corneocyte binding assay was optimized based on previously published methods (67–70), with modifications. For optimization, the effects of time and bacterial concentration were tested on corneocytes collected on 22-mm-diameter adhesive discs (D100 D-Squame standard sampling discs; CuDerm Corp.). Briefly, S. epidermidis 1457 cells grown to an exponential phase (OD_600_, ∼0.7) were harvested, suspended in sterile PBS, and adjusted to an OD_600_ of 0.15, 1.0, or 1.5. An OD_600_ of 0.15 was found to correlate with ∼10^7^ CFU/ml, an OD_600_ of 1.0 with ∼10^8^ CFU/ml, and an OD_600_ of 1.5 with ∼10^9^ CFU/ml. Each corneocyte sample disc was inoculated with one of three concentrations of bacterial suspension and incubated for one of three different time periods (45, 90, or 180 min). For incubation, the discs were placed with the corneocyte surface up in a small petri dish, and 500 μl of each bacterial suspension was placed over the corneocyte layer. Following incubation, the samples were rinsed with PBS 3 times by pipetting and stained with crystal violet for 10 s. Excess stain was removed by rinsing with running water for 10 s, and the discs were then attached to glass microscope slides using transparent glue. The slides were allowed to air dry prior to microscopic examination using light microscopy. An OD_600_ of 0.15 was the chosen bacterial suspension concentration for incubation, due to visibly less clumping observed at this OD. Similarly, 45 min was chosen for the incubation, as 90- and 180-min incubation times resulted in more clumping.

### Corneocyte binding assay.

Overnight cultures of strains transduced with the green fluorescent protein (GFP)-expressing plasmid pCM29 were grown in 5 ml TSB containing 10 μg/ml chloramphenicol with shaking at 250 rpm at 37°C for 16 h. Fresh TSB (12.5 ml in a 125-ml flask) was inoculated at an OD_600_ of 0.1, and the cultures were grown to the exponential phase at 37°C with shaking at 250 rpm until they reached an OD_600_ of ∼0.7. Cells were collected by centrifugation at 5,000 rpm for 5 min at 4°C, and the pellet was washed three times with 1 ml 1× PBS (Dulbecco’s PBS, no calcium, no magnesium). The final bacterial suspension was adjusted to an OD_600_ of 0.15, corresponding to ∼10^7^ CFU/ml in PBS. A 300-μl aliquot of the OD-adjusted suspension was pipetted onto D232 D-Squame discs with adhered corneocytes to form a meniscus and incubated at 37°C for 45 min in a moist chamber. After incubation, the discs were washed with 1× PBS three times by vigorous pipetting and air dried. A cover slip was placed for imaging using confocal laser scanning microscopy.

### Binding inhibition assays.

For binding inhibition assays using antibodies, 300 μl of exponential-phase bacteria (grown and harvested as above), adjusted to an OD_600_ of 0.15, was preincubated with 1:10,000 dilution of anti-Aap A-domain (anti-AapA) or anti-Aap B-domain (anti-AapB) antisera (kindly provided by Holger Rohde) in PBS for 1 h at 37°C. In addition, further dilutions of the anti-AapA antibody were used at 1:10,000, 1:20,000, 1:40,000, 1:80,000, and 1:160,000. Further anti-AapB antibody dilutions were also used at 1:625, 1:1,250, 1:2,500, 1:5,000, and 1:10,000. These additional dilutions of both anti-AapA and anti-AapB antibody were added to 300 μl of bacterial cell suspension for 1 h at 37°C. For anti-polysaccharide intercellular adhesin (PIA), a 1:2,500 dilution was used. The corneocyte binding assay was performed thereafter, as described above. For the blocking assay using recombinant proteins, corneocyte-containing discs were preincubated for 20 min with 300 μl each recombinant Aap domain proteins diluted in PBS to give final concentrations of 0, 0.25, 0.5, 1.0, 5.0, and 10 μM for each protein. Subsequently, the corneocyte binding assay was performed with addition of bacteria to the suspension at a final OD_600_ of 0.15.

Deglycosylation of corneocyte surfaces was performed in which each corneocyte disc was incubated with one of the following deglycosylases—PNGaseF, *O*-glycosidase, α2-3,6,8 neuraminidase, β1-4 galactosidase S, α1-3,4 fucosidase, β-*N*-acetylglucosaminidase S, or α1-2,3 mannosidase (NEB)—for 24 h in a 37°C moist chamber, according to the manufacturer’s instructions. This was followed by complete removal of the incubated enzymes, washing with PBS, and performing the corneocyte binding assay, as described above.

Inhibition of bacterial binding using antibodies against cytokeratin-10 and loricrin was performed, in which corneocytes were preincubated separately with 10 μg/ml rabbit polyclonal anti-cytokeratin-10 IgG (Abcam) and rabbit polyclonal anti-loricrin IgG (Abcam) antibodies for 1 h at 37°C in blocking buffer (1% bovine serum albumin [BSA]) prior to incubation with bacteria.

### Image acquisition and bacterial counting after corneocyte binding assay.

Images were acquired by confocal laser scanning microscopy using an LSM 710 confocal microscope (Zeiss; Jena, Germany). GFP-expressing bacterial cells were observed with an oil immersion objective (Plan-Apochromat 63×/1.40 oil differential interference contrast) using a maximum excitation wavelength (λ_ex_) of 488 nm and a maximum emission wavelength (λ_em_) of 542 nm. Additionally, the nonlabeled corneocyte sheets from the same view field were simultaneously imaged using phase contrast optics. For quantification of adherent bacteria on each disc, 10 images of 89.8 μm × 89.8 μm microscopic fields containing unlabeled confluent corneocytes were captured at 63 × 1.5 magnification, superimposing both the fluorescence and phase contrast image. These fields were randomly selected, and any field in which corneocytes were either not confluent or for which the entire area could not be brought into sharp focus was rejected. In the case of rhodamine-labeled corneocytes, an λ_ex_ of 553 nm and an λ_em_ 627 nm were used for visualization.

Quantitative analysis of the number of adherent bacteria was performed using ImageJ v. 10.0 (NIH, Bethesda, MD). The images captured by the fluorescence channel, which contained the GFP signals (GF- expressing bacteria), were converted from RGB to grayscale in ImageJ. For automated identification of bacteria in each image, thresholding of the image was accomplished by filtering within the hue-saturation-brightness (HSB) color space using the default mode. Subsequently, image noise was removed using “despeckle,” brightness and contrast adjusted to make the bacterial cells more distinguishable from the background. The image was converted to a mask based on the set threshold to produce binary images with inverted lookup tables (LUTs), and “watershed” was used to create segmentation from one another in the case of clustered bacterial cells. The identified bacterial cells were finally counted using “analyze particles.” The pixel counts for each circular area, as well as the average clump size within the area, were automatically entered into spreadsheet format by the software. For each strain tested, the number of adherent bacteria from all of the fields was plotted using GraphPad Prism 8. The mean number of adherent bacteria for each mutant was calculated along with standard error of the mean (SEM). Comparisons between individual mutants were performed by two-tailed unpaired Student’s *t* test with 95% confidence intervals. A *P* value of <0.05 was considered to be significant in each case.

## References

[1] Candi E, Schmidt R, Melino G. 2005. The cornified envelope: a model of cell death in the skin. Nat Rev Mol Cell Biol 6:328–340. doi:10.1038/nrm1619. 15803139

[2] Stacy A, Belkaid Y. 2019. Microbial guardians of skin health. Science 363:227–228. doi:10.1126/science.aat4326. 30655428

[3] Byrd AL, Belkaid Y, Segre JA. 2018. The human skin microbiome. Nat Rev Microbiol 16:143–155. doi:10.1038/nrmicro.2017.157. 29332945

[4] Grice EA, Segre JA. 2011. The skin microbiome. Nat Rev Microbiol 9:244–253. doi:10.1038/nrmicro2537. 21407241 PMC3535073

[5] Belkaid Y, Tamoutounour S. 2016. The influence of skin microorganisms on cutaneous immunity. Nat Rev Immunol 16:353–366. doi:10.1038/nri.2016.48. 27231051

[6] Kong HH, Oh J, Deming C, Conlan S, Grice EA, Beatson MA, Nomicos E, Polley EC, Komarow HD, Program NCS, Murray PR, Turner ML, Segre JA. 2012. Temporal shifts in the skin microbiome associated with disease flares and treatment in children with atopic dermatitis. Genome Res 22:850–859. doi:10.1101/gr.131029.111. 22310478 PMC3337431

[7] Costello EK, Lauber CL, Hamady M, Fierer N, Gordon JI, Knight R. 2009. Bacterial community variation in human body habitats across space and time. Science 326:1694–1697. doi:10.1126/science.1177486. 19892944 PMC3602444

[8] Grice EA, Kong HH, Conlan S, Deming CB, Davis J, Young AC, Program NCS, Bouffard GG, Blakesley RW, Murray PR, Green ED, Turner ML, Segre JA. 2009. Topographical and temporal diversity of the human skin microbiome. Science 324:1190–1192. doi:10.1126/science.1171700. 19478181 PMC2805064

[9] Iwase T, Uehara Y, Shinji H, Tajima A, Seo H, Takada K, Agata T, Mizunoe Y. 2010. *Staphylococcus epidermidis* Esp inhibits *Staphylococcus aureus* biofilm formation and nasal colonization. Nature 465:346–349. doi:10.1038/nature09074. 20485435

[10] Sugimoto S, Iwamoto T, Takada K, Okuda K, Tajima A, Iwase T, Mizunoe Y. 2013. *Staphylococcus epidermidis* Esp degrades specific proteins associated with *Staphylococcus aureus* biofilm formation and host-pathogen interaction. J Bacteriol 195:1645–1655. doi:10.1128/JB.01672-12. 23316041 PMC3624567

[11] Zipperer A, Konnerth MC, Laux C, Berscheid A, Janek D, Weidenmaier C, Burian M, Schilling NA, Slavetinsky C, Marschal M, Willmann M, Kalbacher H, Schittek B, Brotz-Oesterhelt H, Grond S, Peschel A, Krismer B. 2016. Human commensals producing a novel antibiotic impair pathogen colonization. Nature 535:511–516. doi:10.1038/nature18634. 27466123

[12] Nakatsuji T, Chen TH, Narala S, Chun KA, Two AM, Yun T, Shafiq F, Kotol PF, Bouslimani A, Melnik AV, Latif H, Kim JN, Lockhart A, Artis K, David G, Taylor P, Streib J, Dorrestein PC, Grier A, Gill SR, Zengler K, Hata TR, Leung DY, Gallo RL. 2017. Antimicrobials from human skin commensal bacteria protect against *Staphylococcus aureus* and are deficient in atopic dermatitis. Sci Transl Med 9:eaah4680. doi:10.1126/scitranslmed.aah4680. 28228596 PMC5600545

[13] Naik S, Bouladoux N, Wilhelm C, Molloy MJ, Salcedo R, Kastenmuller W, Deming C, Quinones M, Koo L, Conlan S, Spencer S, Hall JA, Dzutsev A, Kong H, Campbell DJ, Trinchieri G, Segre JA, Belkaid Y. 2012. Compartmentalized control of skin immunity by resident commensals. Science 337:1115–1119. doi:10.1126/science.1225152. 22837383 PMC3513834

[14] Naik S, Bouladoux N, Linehan JL, Han SJ, Harrison OJ, Wilhelm C, Conlan S, Himmelfarb S, Byrd AL, Deming C, Quinones M, Brenchley JM, Kong HH, Tussiwand R, Murphy KM, Merad M, Segre JA, Belkaid Y. 2015. Commensal-dendritic-cell interaction specifies a unique protective skin immune signature. Nature 520:104–108. doi:10.1038/nature14052. 25539086 PMC4667810

[15] Stones DH, Krachler AM. 2016. Against the tide: the role of bacterial adhesion in host colonization. Biochem Soc Trans 44:1571–1580. doi:10.1042/BST20160186. 27913666 PMC5134996

[16] Feuillie C, Vitry P, McAleer MA, Kezic S, Irvine AD, Geoghegan JA, Dufrene YF. 2018. Adhesion of *Staphylococcus aureus* to corneocytes from atopic dermatitis patients is controlled by natural moisturizing factor levels. mBio 9:e01184-18. doi:10.1128/mBio.01184-18. 30108169 PMC6094479

[17] Fleury OM, McAleer MA, Feuillie C, Formosa-Dague C, Sansevere E, Bennett DE, Towell AM, McLean WHI, Kezic S, Robinson DA, Fallon PG, Foster TJ, Dufrene YF, Irvine AD, Geoghegan JA. 2017. Clumping factor B promotes adherence of *Staphylococcus aureus* to corneocytes in atopic dermatitis. Infect Immun 85:e00994-16. doi:10.1128/IAI.00994-16. 28373353 PMC5442637

[18] Bitschar K, Staudenmaier L, Klink L, Focken J, Sauer B, Fehrenbacher B, Herster F, Bittner Z, Bleul L, Schaller M, Wolz C, Weber ANR, Peschel A, Schittek B. 2020. *Staphylococcus aureus* skin colonization is enhanced by the interaction of neutrophil extracellular traps with keratinocytes. J Invest Dermatol 140:1054–1065.e4. doi:10.1016/j.jid.2019.10.017. 31857094

[19] Edslev SM, Agner T, Andersen PS. 2020. Skin microbiome in atopic dermatitis. Acta Derm Venereol 100:adv00164. doi:10.2340/00015555-3514. 32419029 PMC9189751

[20] Foster TJ. 2020. Surface proteins of *Staphylococcus epidermidis*. Front Microbiol 11:1829. doi:10.3389/fmicb.2020.01829. 32849430 PMC7403478

[21] Speziale P, Pietrocola G, Foster TJ, Geoghegan JA. 2014. Protein-based biofilm matrices in staphylococci. Front Cell Infect Microbiol 4:171. doi:10.3389/fcimb.2014.00171. 25540773 PMC4261907

[22] Foster TJ, Geoghegan JA, Ganesh VK, Hook M. 2014. Adhesion, invasion and evasion: the many functions of the surface proteins of *Staphylococcus aureus*. Nat Rev Microbiol 12:49–62. doi:10.1038/nrmicro3161. 24336184 PMC5708296

[23] Rohde H, Burandt EC, Siemssen N, Frommelt L, Burdelski C, Wurster S, Scherpe S, Davies AP, Harris LG, Horstkotte MA, Knobloch JK, Ragunath C, Kaplan JB, Mack D. 2007. Polysaccharide intercellular adhesin or protein factors in biofilm accumulation of *Staphylococcus epidermidis* and *Staphylococcus aureus* isolated from prosthetic hip and knee joint infections. Biomaterials 28:1711–1720. doi:10.1016/j.biomaterials.2006.11.046. 17187854

[24] Schaeffer CR, Hoang TN, Sudbeck CM, Alawi M, Tolo IE, Robinson DA, Horswill AR, Rohde H, Fey PD. 2016. Versatility of biofilm matrix molecules in *Staphylococcus epidermidis* clinical isolates and importance of polysaccharide intercellular adhesin expression during high shear stress. mSphere 1:e00165-16. doi:10.1128/mSphere.00165-16. 27747298 PMC5064449

[25] Gotz F. 2002. *Staphylococcus* and biofilms. Mol Microbiol 43:1367–1378. doi:10.1046/j.1365-2958.2002.02827.x. 11952892

[26] Schumacher-Perdreau F, Heilmann C, Peters G, Gotz F, Pulverer G. 1994. Comparative analysis of a biofilm-forming *Staphylococcus epidermidis* strain and its adhesion-positive, accumulation-negative mutant M7. FEMS Microbiol Lett 117:71–78. doi:10.1111/j.1574-6968.1994.tb06744.x. 8181711

[27] Hussain M, Herrmann M, von Eiff C, Perdreau-Remington F, Peters G. 1997. A 140-kilodalton extracellular protein is essential for the accumulation of *Staphylococcus epidermidis* strains on surfaces. Infect Immun 65:519–524. doi:10.1128/iai.65.2.519-524.1997. 9009307 PMC176090

[28] Banner MA, Cunniffe JG, Macintosh RL, Foster TJ, Rohde H, Mack D, Hoyes E, Derrick J, Upton M, Handley PS. 2007. Localized tufts of fibrils on *Staphylococcus epidermidis* NCTC 11047 are comprised of the accumulation-associated protein. J Bacteriol 189:2793–2804. doi:10.1128/JB.00952-06. 17277069 PMC1855787

[29] Conrady DG, Wilson JJ, Herr AB. 2013. Structural basis for Zn^2+^-dependent intercellular adhesion in staphylococcal biofilms. Proc Natl Acad Sci U S A 110:E202–11. doi:10.1073/pnas.1208134110. 23277549 PMC3549106

[30] Gruszka DT, Wojdyla JA, Bingham RJ, Turkenburg JP, Manfield IW, Steward A, Leech AP, Geoghegan JA, Foster TJ, Clarke J, Potts JR. 2012. Staphylococcal biofilm-forming protein has a contiguous rod-like structure. Proc Natl Acad Sci U S A 109:E1011–E1018. doi:10.1073/pnas.1119456109. 22493247 PMC3340054

[31] Bowden MG, Chen W, Singvall J, Xu Y, Peacock SJ, Valtulina V, Speziale P, Höök M. 2005. Identification and preliminary characterization of cell-wall-anchored proteins of *Staphylococcus epidermidis*. Microbiology 151:1453–1464. doi:10.1099/mic.0.27534-0. 15870455

[32] Conlon BP, Geoghegan JA, Waters EM, McCarthy H, Rowe SE, Davies JR, Schaeffer CR, Foster TJ, Fey PD, O’Gara JP. 2014. Role for the A domain of unprocessed accumulation-associated protein (Aap) in the attachment phase of the *Staphylococcus epidermidis* biofilm phenotype. J Bacteriol 196:4268–4275. doi:10.1128/JB.01946-14. 25266380 PMC4248850

[33] Schaeffer CR, Woods KM, Longo GM, Kiedrowski MR, Paharik AE, Buttner H, Christner M, Boissy RJ, Horswill AR, Rohde H, Fey PD. 2015. Accumulation-associated protein enhances *Staphylococcus epidermidis* biofilm formation under dynamic conditions and is required for infection in a rat catheter model. Infect Immun 83:214–226. doi:10.1128/IAI.02177-14. 25332125 PMC4288872

[34] Paharik AE, Kotasinska M, Both A, Hoang TN, Buttner H, Roy P, Fey PD, Horswill AR, Rohde H. 2017. The metalloprotease SepA governs processing of accumulation-associated protein and shapes intercellular adhesive surface properties in *Staphylococcus epidermidis*. Mol Microbiol 103:860–874. doi:10.1111/mmi.13594. 27997732 PMC5480372

[35] Rohde H, Burdelski C, Bartscht K, Hussain M, Buck F, Horstkotte MA, Knobloch JK, Heilmann C, Herrmann M, Mack D. 2005. Induction of *Staphylococcus epidermidis* biofilm formation via proteolytic processing of the accumulation-associated protein by staphylococcal and host proteases. Mol Microbiol 55:1883–1895. doi:10.1111/j.1365-2958.2005.04515.x. 15752207

[36] Bateman A, Holden MT, Yeats C. 2005. The G5 domain: a potential *N*-acetylglucosamine recognition domain involved in biofilm formation. Bioinformatics 21:1301–1303. doi:10.1093/bioinformatics/bti206. 15598841

[37] Conrady DG, Brescia CC, Horii K, Weiss AA, Hassett DJ, Herr AB. 2008. A zinc-dependent adhesion module is responsible for intercellular adhesion in staphylococcal biofilms. Proc Natl Acad Sci U S A 105:19456–19461. doi:10.1073/pnas.0807717105. 19047636 PMC2592360

[38] Macintosh RL, Brittan JL, Bhattacharya R, Jenkinson HF, Derrick J, Upton M, Handley PS. 2009. The terminal A domain of the fibrillar accumulation-associated protein (Aap) of *Staphylococcus epidermidis* mediates adhesion to human corneocytes. J Bacteriol 191:7007–7016. doi:10.1128/JB.00764-09. 19749046 PMC2772481

[39] Trivedi S, Uhlemann AC, Herman-Bausier P, Sullivan SB, Sowash MG, Flores EY, Khan SD, Dufrene YF, Lowy FD. 2017. The surface protein SdrF mediates *Staphylococcus epidermidis* adherence to keratin. J Infect Dis 215:1846–1854. doi:10.1093/infdis/jix213. 28482041 PMC5853823

[40] Hoang TM, Zhou C, Lindgren JK, Galac MR, Corey B, Endres JE, Olson ME, Fey PD. 2019. Transcriptional regulation of *icaADBC* by both IcaR and TcaR in *Staphylococcus epidermidis*. J Bacteriol 201:e00524-18. doi:10.1128/JB.00524-18. PMC6398268 30602488

[41] Mazmanian SK, Ton-That H, Schneewind O. 2001. Sortase-catalysed anchoring of surface proteins to the cell wall of *Staphylococcus aureus*. Mol Microbiol 40:1049–1057. doi:10.1046/j.1365-2958.2001.02411.x. 11401711

[42] Roche FM, Meehan M, Foster TJ. 2003. The *Staphylococcus aureus* surface protein SasG and its homologues promote bacterial adherence to human desquamated nasal epithelial cells. Microbiology 149:2759–2767. doi:10.1099/mic.0.26412-0. 14523109

[43] Yarawsky AE, English LR, Whitten ST, Herr AB. 2017. The Proline/glycine-rich region of the biofilm adhesion protein Aap forms an extended stalk that resists compaction. J Mol Biol 429:261–279. doi:10.1016/j.jmb.2016.11.017. 27890783 PMC5363081

[44] Varki A. 2011. Evolutionary forces shaping the Golgi glycosylation machinery: why cell surface glycans are universal to living cells. Cold Spring Harb Perspect Biol 3:a005462. doi:10.1101/cshperspect.a005462. 21525513 PMC3098673

[45] Schnaar RL. 2015. Glycans and glycan-binding proteins in immune regulation: a concise introduction to glycobiology for the allergist. J Allergy Clin Immunol 135:609–615. doi:10.1016/j.jaci.2014.10.057. 25649080 PMC4355172

[46] Tang L, Chen X, Zhang X, Guo Y, Su J, Zhang J, Peng C, Chen X. 2019. *N*-Glycosylation in progression of skin cancer. Med Oncol 36. doi:10.1007/s12032-019-1270-4. 31037368

[47] Danzberger J, Donovan M, Rankl C, Zhu R, Vicic S, Baltenneck C, Enea R, Hinterdorfer P, Luengo GS. 2018. Glycan distribution and density in native skin’s stratum corneum. Skin Res Technol 24:450–458. doi:10.1111/srt.12453. 29417655 PMC6446803

[48] Crosby HA, Schlievert PM, Merriman JA, King JM, Salgado-Pabon W, Horswill AR. 2016. The *Staphylococcus aureus* global regulator MgrA modulates clumping and virulence by controlling surface protein expression. PLoS Pathog 12:e1005604. doi:10.1371/journal.ppat.1005604. 27144398 PMC4856396

[49] Nakatsuji T, Chen TH, Butcher AM, Trzoss LL, Nam SJ, Shirakawa KT, Zhou W, Oh J, Otto M, Fenical W, Gallo RL. 2018. A commensal strain of *Staphylococcus epidermidis* protects against skin neoplasia. Sci Adv 4:eaao4502. doi:10.1126/sciadv.aao4502. 29507878 PMC5834004

[50] Varki A, Cummings RD, Esko JD, Stanley P, Hart GW, Aebi M, Darvill AG, Kinoshita T, Packer NH, Prestegard JH, Schnaar RL, Seeberger PH (ed). 2015. Essentials of glycobiology. Cold Spring Harbor Laboratory Press, Cold Spring Harbor, NY. 27010055

[51] Ilver D, Johansson P, Miller-Podraza H, Nyholm PG, Teneberg S, Karlsson KA. 2003. Bacterium-host protein-carbohydrate interactions. Methods Enzymol 363:134–157. doi:10.1016/S0076-6879(03)01049-8. 14579573

[52] Gonzalez T, Stevens ML, Kyzy AB, Alarcon R, He H, Kroner JW, Spagna D, Grashel B, Sidler E, Martin LJ, Biagini Myers JM, Hershey K, Herr AB. 2021. Biofilm propensity of *Staphylococcus aureus* skin isolates is associated with increased atopic dermatitis severity and barrier dysfunction in the MPAACH pediatric cohort. Allergy 76:302–312. doi:10.1111/all.14489. 32640045 PMC8561741

[53] Kwiecinski JM, Crosby HA, Valotteau C, Hippensteel JA, Nayak MK, Chauhan AK, Schmidt EP, Dufrene YF, Horswill AR. 2019. *Staphylococcus aureus* adhesion in endovascular infections is controlled by the ArlRS-MgrA signaling cascade. PLoS Pathog 15:e1007800. doi:10.1371/journal.ppat.1007800. 31116795 PMC6548404

[54] Varki A. 2017. Biological roles of glycans. Glycobiology 27:3–49. doi:10.1093/glycob/cww086. 27558841 PMC5884436

[55] Mack D, Siemssen N, Laufs R. 1992. Parallel induction by glucose of adherence and a polysaccharide antigen specific for plastic-adherent *Staphylococcus epidermidis*: evidence for functional relation to intercellular adhesion. Infect Immun 60:2048–2057. doi:10.1128/iai.60.5.2048-2057.1992. 1314224 PMC257114

[56] Studier FW. 2005. Protein production by auto-induction in high density shaking cultures. Protein Expr Purif 41:207–234. doi:10.1016/j.pep.2005.01.016. 15915565

[57] Bose JL, Fey PD, Bayles KW. 2013. Genetic tools to enhance the study of gene function and regulation in *Staphylococcus aureus*. Appl Environ Microbiol 79:2218–2224. doi:10.1128/AEM.00136-13. 23354696 PMC3623228

[58] Projan SJ, Archer GL. 1989. Mobilization of the relaxable *Staphylococcus aureus* plasmid pC221 by the conjugative plasmid pGO1 involves three pC221 loci. J Bacteriol 171:1841–1845. doi:10.1128/jb.171.4.1841-1845.1989. 2703461 PMC209830

[59] Winstel V, Kuhner P, Krismer B, Peschel A, Rohde H. 2015. Transfer of plasmid DNA to clinical coagulase-negative staphylococcal pathogens by using a unique bacteriophage. Appl Environ Microbiol 81:2481–2488. doi:10.1128/AEM.04190-14. 25616805 PMC4357934

[60] Maliszewski KL, Nuxoll AS. 2014. Use of electroporation and conjugative mobilization for genetic manipulation of *Staphylococcus epidermidis*. Methods Mol Biol 1106:125–134. doi:10.1007/978-1-62703-736-5_11. 24222461

[61] Olson ME, Horswill AR. 2014. Bacteriophage transduction in *Staphylococcus epidermidis*. Methods Mol Biol 1106:167–172. doi:10.1007/978-1-62703-736-5_15. 24222465 PMC4711990

[62] Bose JL. 2014. Genetic manipulation of staphylococci. Methods Mol Biol 1106:101–111. doi:10.1007/978-1-62703-736-5_8. 24222458

[63] Handke LD, Slater SR, Conlon KM, O’Donnell ST, Olson ME, Bryant KA, Rupp ME, O'Gara JP, Fey PD. 2007. SigmaB and SarA independently regulate polysaccharide intercellular adhesin production in *Staphylococcus epidermidis*. Can J Microbiol 53:82–91. doi:10.1139/w06-108. 17496953

[64] Yajjala VK, Widhelm TJ, Endres JL, Fey PD, Bayles KW. 2016. Generation of a transposon mutant library in *Staphylococcus aureus* and *Staphylococcus epidermidis* using *bursa aurealis*. Methods Mol Biol 1373:103–110. doi:10.1007/7651_2014_189. 25682373

[65] Goering RV, Fey PD. 2014. Pulsed field gel electrophoresis of *Staphylococcus epidermidis*. Methods Mol Biol 1106:55–60. doi:10.1007/978-1-62703-736-5_4. 24222454

[66] Hirao T, Denda M, Takahashi M. 2001. Identification of immature cornified envelopes in the barrier-impaired epidermis by characterization of their hydrophobicity and antigenicities of the components. Exp Dermatol 10:35–44. doi:10.1034/j.1600-0625.2001.100105.x. 11168578

[67] Griffeth GC, Callori N, Rankin SC, Boston RC, Morris DO. 2012. Optimization of a *Staphylococcus aureus* adhesion assay for equine corneocytes. Vet Dermatol 23:57–60.e13. doi:10.1111/j.1365-3164.2011.01012.x. 21992593 PMC3253182

[68] Lu YF, McEwan NA. 2007. Staphylococcal and micrococcal adherence to canine and feline corneocytes: quantification using a simple adhesion assay. Vet Dermatol 18:29–35. doi:10.1111/j.1365-3164.2007.00567.x. 17222237

[69] Latronico F, Moodley A, Nielsen SS, Guardabassi L. 2014. Enhanced adherence of methicillin-resistant *Staphylococcus pseudintermedius* sequence type 71 to canine and human corneocytes. Vet Res 45:70. doi:10.1186/1297-9716-45-70. 24957656 PMC4087241

[70] Simou C, Hill PB, Forsythe PJ, Thoday KL. 2005. Species specificity in the adherence of staphylococci to canine and human corneocytes: a preliminary study. Vet Dermatol 16:156–161. doi:10.1111/j.1365-3164.2005.00452.x. 15960628

