# Supplementary material for: Glycan-Dependent Corneocyte Adherence of Staphylococcus epidermidis Mediated by the Lectin Subdomain of Aap
Source: mBio. 2021 Jul 13;12(4):e02908-20. doi: 10.1128/mBio.02908-20 (PMC8406310; doi:10.1128/mBio.02908-20)
Supplement: FIG S2 [file mbio.02908-20-sf002.pdf]

Figure S2

A

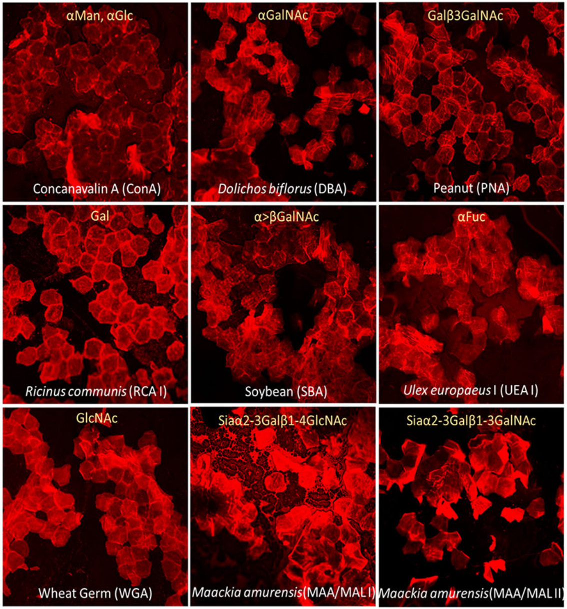

Figure S2. Detection of rhodamine labelled lectins on the surface of corneocytes. Patterns of glycan distribution, observed by confocal microscopy at a magnification of 40 x 0.6 (A) or 63 x 1.5 (B) . Lipid-rich layers of the stratum corneum were not removed in order to obtain a realistic picture of glycan localization on the surface of native corneocytes. Abbreviations: Man: D-mannose; Glc: D-glucose; Gal: D-Galactose; Fuc: L-Fucose; GlcNAc: *N*-acetylglucosamine; GalNAc: *N*-acetylgalactosamine; Sia: sialic acid (*N*-acetylneuraminic acid). Labelling of the corneocyte periphery with beaded appearance was obtained very prominently with RCA I with specificity to β-D-galactose (Gal). Peripheral labelling was also obtained with ConA, PNA, SBA and WGA, with specificities for α-D-mannose (Man) and α-D-glucose (Glc), galactose β3-linked *N*-acetylgalactosamine (Galβ3GalNAc), α>βGalNAc and *N*-acetylglucosamine (GlcNAc), respectively. A Fine netlike distribution was observed with DBA, PNA, UEA, and MAA/MAL, with specificities for α-GalNAc, Galβ3GalNAc, α-L-fucose (Fuc) and α-(2,3) linked sialic acid (Sia), with very prominent signature observed using MAA/MAL I specific for Siaα2-3Galβ1-4GlcNAc. Additional diffused labelling dispersed over the corneocyte surface was observed for all the above lectins, except RCA I. The labelling was weak and uniformly distributed over the corneocyte surface with SNA/EBL I, which recognizes α-(2,6) linked Sia (image not shown).

Figure S2

B

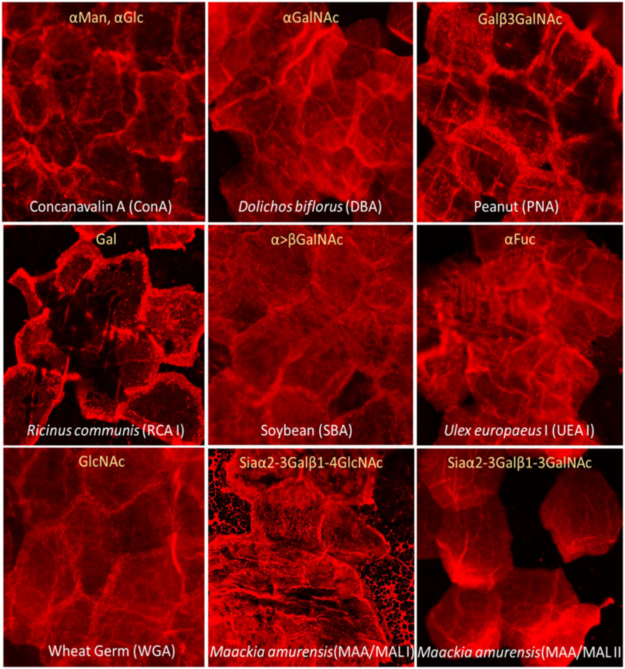

Magnification: 63 x 1.5

89.8 µm x 89.8 µm sections
