# Supplementary material for: Glycan-Dependent Corneocyte Adherence of Staphylococcus epidermidis Mediated by the Lectin Subdomain of Aap
Source: mBio. 2021 Jul 13;12(4):e02908-20. doi: 10.1128/mBio.02908-20 (PMC8406310; doi:10.1128/mBio.02908-20)
Supplement: TABLE S3 [file mbio.02908-20-st003.docx]

**Table S3:** Sequence homology of *S. epidermidis* 1457 Aap and its Lectin domain with other staphylococcal skin commensals

| Accession number | Annotated as | Overall Aap sequence | | Aap lectin domain sequence | |
| --- | --- | --- | --- | --- | --- |
|  |  | **Query cover (%)** | **Identity (%)** | **Query cover (%)** | **Identity (%)** |
| ***S. hominis*** | | | | | |
| WP_172473849.1 | E domain-containing protein | 70 | 75 | 100 | 79 |
| WP_165488913.1 | E domain-containing protein | 70 | 75 | 100 | 79 |
| PAL08538.1 | Hypothetical protein B8W90_09180 | 88 | 67 | 100 | 79 |
| AUW64022.1 | Hypothetical protein AL495_10410 | 63 | 71 | 55 | 51 |
| OJH00147.1 | Hypothetical protein BL313_11585 | 88 | 54 | 89 | 51 |
| WP_152667463.1 | E domain-containing protein | 84 | 55 | 89 | 51 |
| TRL61691.1 | Accumulation-associated protein | 68 | 80 | 100 | 79 |
| WP_158260991.1 | E domain-containing protein | 90 | 65 | 100 | 73 |
| WP_158260981.1 | E domain-containing protein | 90 | 65 | 100 | 76 |
| WP_107642678.1 | E domain-containing protein | 90 | 68 | 100 | 76 |
| WP_107622890.1 | E domain-containing protein | 99 | 58 | 100 | 76 |
| WP_107636353.1 | E domain-containing protein | 91 | 58 | 100 | 76 |
| WP_158260973.1 | E domain-containing protein | 90 | 58 | 100 | 76 |
| WP_100476041.1 | E domain-containing protein | 89 | 52 | 89 | 51 |
| EHR90443.1 | Surface protein | 75 | 56 | 89 | 51 |
| OUL44712.1 | Hypothetical protein B2G94_11085 | 76 | 55 | 89 | 50 |
| WP_145436181.1 | YSIRK-type signal peptide-containing protein | 62 | 58 | 100 | 78 |
| TRL30723.1 | Accumulation-associated protein | 70 | 78 | 100 | 79 |
| WP_100442634.1 | E domain-containing protein | 64 | 66 | 66 | 51 |
| RLY88401.1 | YSIRK-type signal peptide-containing protein | 88 | 58 | 100 | 78 |
| EEK11249.1 | G5 domain protein | 72 | 63 | 100 | 76 |
| WP_172848048.1 | G5 domain protein | 85 | 54 | 100 | 76 |
| WP_119626219.1 | YSIRK-type signal peptide-containing protein | 55 | 63 | 100 | 76 |
| WP_171991299.1 | E domain-containing protein | 77 | 47 | 89 | 51 |
| WP_069723882.1 | E domain-containing protein | 68 | 53 | 89 | 51 |
| ***S. haemolyticus*** | | | | | |

| WP_080366921.1 | E domain-containing protein | 70 | 80 | 100 | 79 |
| --- | --- | --- | --- | --- | --- |
| WP_080367133.1 | E domain-containing protein | 70 | 79 | 100 | 79 |
| WP_080365735.1 | E domain-containing protein | 70 | 79 | 100 | 79 |
| WP_080364131.1 | E domain-containing protein | 70 | 79 | 100 | 79 |
| WP_154884060.1 | E domain-containing protein | 90 | 63 | 100 | 79 |
| RJG29875.1 | Accumulation-associated protein | 70 | 76 | 100 | 79 |
| WP_053028418.1 | E domain-containing protein | 70 | 79 | 100 | 79 |
| WP_154392047.1 | E domain-containing protein | 86 | 69 | 100 | 79 |
| WP_080366774.1 | E domain-containing protein | 70 | 79 | 100 | 79 |
| WP_137028787.1 | E domain-containing protein | 84 | 51 | 89 | 51 |
| WP_136991714.1 | E domain-containing protein | 84 | 50 | 89 | 51 |
| RSZ22489.1 | Accumulation-associated protein | 70 | 77 | 100 | 79 |
| WP_154393694.1 | E domain-containing protein | 88 | 48 | 90 | 51 |
| RJG22250.1 | Accumulation-associated protein | 71 | 55 | 89 | 51 |
| WP_154391785.1 | E domain-containing protein | 65 | 61 | 91 | 50 |
| WP_157047532.1 | E domain-containing protein | 90 | 45 | 89 | 51 |
| WP_053028425.1 | E domain-containing protein | 90 | 44 | 89 | 50 |
| WP_157042244.1 | E domain-containing protein | 90 | 44 | 89 | 50 |
| WP_157037706.1 | E domain-containing protein | 90 | 44 | 89 | 50 |
| RFU02382.1 | Hypothetical protein DT247_11575 | 82 | 46 | 89 | 50 |
| TPX78759.1 | Accumulation-associated protein | 70 | 50 | 91 | 50 |
| WP_117276971.1 | E domain-containing protein | 83 | 42 | 89 | 50 |
| WP_154393492.1 | E domain-containing protein | 78 | 42 | 89 | 50 |
| WP_154838456.1 | E domain-containing protein | 64 | 55 | 66 | 51 |
| WP_161593951.1 | YSIRK-type signal peptide-containing protein | 71 | 41 | 89 | 51 |
| WP_145464694.1 | YSIRK-type signal peptide-containing protein | 71 | 39 | 89 | 51 |
| WP_154230755.1 | YSIRK-type signal peptide-containing protein | 65 | 38 | 89 | 51 |
| WP_100479701.1 | E domain-containing protein | 87 | 42 | 89 | 50 |
| ***S. saprophyticus*** | | | | | |
| WP_135092910.1 | E domain-containing protein | 87 | 47 | 99 | 46 |
| WP_156676130.1 | E domain-containing protein | 88 | 45 | 91 | 50 |
| SUM78941.1 | Methicillin-resistant surface protein | 71 | 53 | 91 | 48 |
| ***S. capitis*** | | | | | |
| AKL93117.1 | Accumulation associated protein | 90 | 51 | 91 | 50 |
| EFS16612.1 | Cell wall surface anchor family protein | 70 | 91 | 100 | 94 |
| ***S. warneri*** | | | | | |
| PTI82042.1 | Hypothetical protein BU077_10470 | 71 | 73 | 0 | 0 |
| WP_131541458.1 | E domain-containing protein | 56 | 80 | 0 | 0 |
