# Supplementary figures and images for: Glycan-Dependent Corneocyte Adherence of Staphylococcus epidermidis Mediated by the Lectin Subdomain of Aap
Source: mBio. 2021 Jul 13;12(4):e02908-20. doi: 10.1128/mBio.02908-20 (PMC8406310; doi:10.1128/mBio.02908-20)

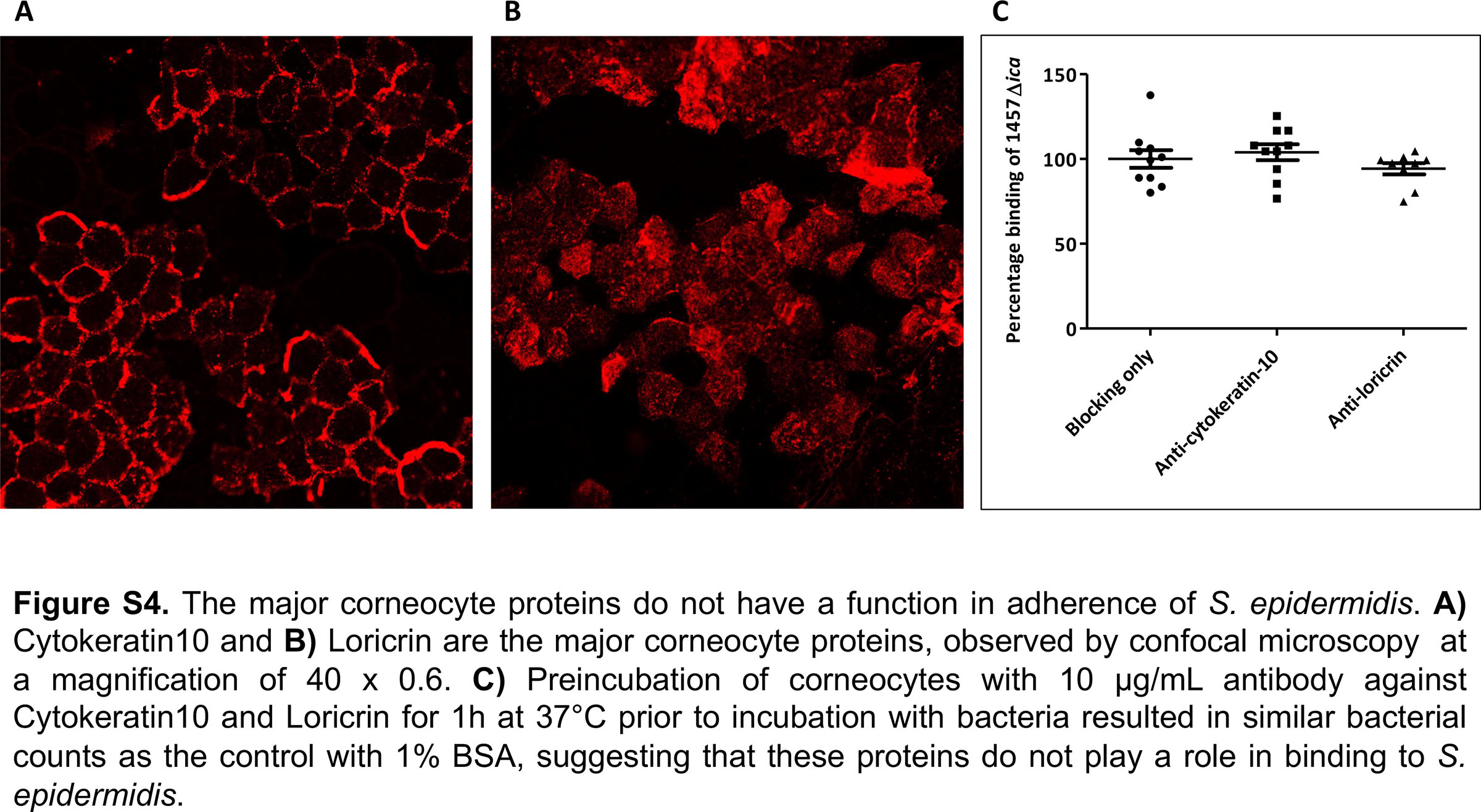

Supplement: FIG S4 [file mbio.02908-20-sf004.jpg]
