# Supplementary material for: Glycan-Dependent Corneocyte Adherence of Staphylococcus epidermidis Mediated by the Lectin Subdomain of Aap
Source: mBio. 2021 Jul 13;12(4):e02908-20. doi: 10.1128/mBio.02908-20 (PMC8406310; doi:10.1128/mBio.02908-20)
Supplement: TABLE S1 [file mbio.02908-20-st001.docx]

**Table S1:** Bacterial strains, plasmids and bacteriophages used in this study.

| Strain | Description | Source or reference |
| --- | --- | --- |
| *Escherichia coli* DH5α | Cloning host. | Invitrogen |
| *E. coli* BL21(DE3) | Protein over-expression host. | Invitrogen |
| *S. aureus* PS187Δ*hsdR* Δ*sauUSI* | Φ187 propagation strain. | ([1](#_ENREF_1)) |
| *S. epidermidis* 1457 | Central venous catheter infection *S. epidermidis* isolate. | ([2](#_ENREF_2)) |
| *S. epidermidis* 1457Δ*ica* | 1457*icaADBC::dhfr*; PIA-negative, Aap-positive; Tmp^R^. | ([3](#_ENREF_3)) |
| *S. epidermidis* 1457Δ*aap* | 1457*aap::tetM*; PIA-positive, Aap-negative; Tet^R^. | ([4](#_ENREF_4)) |
| *S. epidermidis* 1457Δ*ica*Δ*aap* | 1457*icaADBC::dhfr aap::tetM*; PIA negative, Aap negative; Tmp^R^, Tet^R^. | ([4](#_ENREF_4)) |
| *S. epidermidis* 1457Δ*srtA* | 1457*srtA::tetM*; PIA-positive, sortaseA negative; Tet^R^. | This work |
| *S. epidermidis* 1457Δ*ica*Δ*srtA* | 1457*icaADBC::dhfr srtA::tetM*; PIA negative, sortaseA negative; Tmp^R^, Tet^R^. | This work |
| *S. epidermidis* 1457Δ*ica*Δ*sdrF* | 1457*icaADBC::dhfr sdrF::ermC*; PIA negative, Aap positive, SdrF negative; Tmp^R^, Erm^R^. | This work |
| *S. epidermidis* 1457Δ*ica*Δ*aap*Δ*sdrF* | 1457*icaADBC::dhfr aap::tetM sdrF::ermC*; PIA negative, Aap negative, SdrF negative; Tmp^R^, Tet^R^, Erm^R^. | This work |
| *S. epidermidis* 1457Δ*ica aap::A-(rep+lec)* | 1457*icaADBC::dhfr* mutant expressing the complete A domain of Aap; PIA negative; Tmp^R^. | This work |
| *S. epidermidis* 1457Δ*ica aap::B* | 1457*icaADBC::dhfr* mutant expressing B domain of Aap; PIA negative; Tmp^R^. | This work |
| *S. epidermidis* 1457Δ*ica aap::A-rep* | 1457*icaADBC::dhfr* mutant expressing A-repeat region of Aap; PIA negative; Tmp^R^. | This work |
| *S. epidermidis* 1457Δ*ica aap::A-lec* | 1457*icaADBC::dhfr* mutant expressing A-lectin subdomain of Aap; PIA negative; Tmp^R^. | This work |
| *S. epidermidis* 1457Δ*ica aap::A-rep+B* | 1457*icaADBC::dhfr* mutant expressing Aap which has the A-repeat region attached to the B domain; PIA negative; Tmp^R^. | This work |
| *S. epidermidis* 1457Δ*ica aap::A-lec+B* | 1457*icaADBC::dhfr* mutant expressing Aap which has the A-lectin subdomain attached to the B domain; PIA negative; Tmp^R^. | This work |
| *S. epidermidis* 1457Δ*ica aap::A-(rep+lec)+B* | 1457*icaADBC::dhfr* mutant expressing the full-length Aap; PIA negative; Tmp^R^. | This work |
| *S. saprophyticus* 01 | Clinical isolate. | This work |
| *S. haemolyticus* 02 | Clinical isolate. | This work |
| *S. capitis* 03 | Clinical isolate. | This work |
| *S. hominis* 04 | Clinical isolate. | This work |
| *S. simulans* 05 | Clinical isolate. | This work |
| *S. warneri* 06 | Clinical isolate. | This work |
| *S. aureus* MW2 | USA400 community-acquired methicillin-resistant *S. aureus*. | ([5](#_ENREF_5)) |
| *S. aureus* MW2Δ*sasG* (AH3812) | *sasG* negative. | ([6](#_ENREF_6)) |
| *S. aureus* MW2Δ*mgrA* (AH3422) | *mgrA* negative. | ([6](#_ENREF_6)) |
| *S. aureus* MW2Δ*mgrA* Δ*sasG* (AH3989) | *mgrA* negative, *sasG* negative. | ([6](#_ENREF_6)) |

| Plasmid | Description | Source or reference |
| --- | --- | --- |
| pET28a | Protein over-expression vector in *E. coli*; N-terminal His6-tag fusion; Kan^R^. | Novagen |
| pCR2.1 | TA cloning vector; Kan^R^, Amp^R^. | Invitrogen |
| pUC19 | Cloning vector containing MCS; Amp^R^. | ([7](#_ENREF_7)) |
| pROJ6448 | pE194(ts) with pC221 nick site cloned into the unique ClaI site; Erm^R^. | ([8](#_ENREF_8)) |
| pCM29 | GFP fluorescence expression vector; *sarA* P1 promoter driving the sGFP gene; Chl^R^. | ([9](#_ENREF_9)) |
| pJB38 | Temperature sensitive staphylococcal shuttle vector for chromosomal integration; pET194ts oriV, pBR322 oriV; *Pxyl/tetO-secY570* for counter-selection; Chl^R^ Amp^R^. | ([10](#_ENREF_10)) |
| pNF319 | rA_rep+lec_ domain Aap protein over-expression vector; pET28a with *aap* A_rep+lec_ domain; Kan^R^. | This work |
| pNF326 | rA_repeat_ subdomain Aap protein over-expression vector; pET28a with *aap* A-repeat region; Kan^R^. | This work |
| pNF318 | rA_lectin_ subdomain Aap protein over-expression vector; pET28a with *aap* A-lectin subdomain; Kan^R^. | This work |
| pNF79 | pCR2.1 with *srtA* gene and its upstream and downstream region cloned into the TA cloning site. Kan^R^, Amp^R^. | This work |
| pNF82 | pUC19 with *srtA* gene and its upstream and downstream region cloned into the EcoRI site. Amp^R^. | This work |
| pNF107 | pNF82 with *tetM* inserted at the ClaI site in the middle of *srtA*; Tet^R^. Amp^R^. | This work |
| pNF112 | *srtA* allelic replacement vector; pNF107 with pROJ6448 inserted at the PstI site; Tet^R^. Erm^R^, Amp^R^. | This work |
| pNF105 | pGEM with ClaI tailed *tetM* cassette. Tet^R^. Amp^R^. | This work |
| pNF360 | pJB38 with 3’ *sdrF* and downstream fragment inserted in between the XmaI and SalI sites. Chl^R^, Amp^R^. | This work |
| pNF361 | pNF360 with 5’ *sdrF* and upstream fragment inserted in between the SacI and XmaI sites. Chl^R^, Amp^R^. | This work |
| pNF362 | *sdrF* allelic replacement vector; pNF361 with *ermC* inserted at the XmaI site. Erm^R^, Chl^R^, Amp^R^. | This work |
| pNF344 | *aap*A-repeat allelic exchange vector introduced in 1457*icaADBC::dhfr aap::A-lec+B*; pJB38 with [UP+*aap*(SS+A-(rep+lec)] inserted in between the EcoRI and XmaI site; Chl^R^, Amp^R^. | This work |
| pNF348 | *aap*A-(rep+lec) allelic exchange vector; pNF344 with [*aap*(PGR+LPDTG)+DN] inserted in between the XmaI and SalI site; Chl^R^, Amp^R^. | This work |
| pNF334 | pJB38 with [UP+*aap*(SS)] inserted in between the EcoRI and XmaI site; Chl^R^, Amp^R^. | This work |
| pNF343 | *aap*B allelic exchange vector; pNF334 with [*aap*(B+PGR+LPDTG)+DN] inserted in between the XmaI and SalI site; Chl^R^, Amp^R^. | This work |
| pNF346 | pJB38 with [UP+*aap*(SS+A-rep)] inserted in between the EcoRI and XmaI site; Chl^R^, Amp^R^. | This work |
| pNF347 | *aap*A-repeat allelic exchange vector; pNF346 with [*aap*(PGR+LPDTG)+DN] inserted in between the XmaI and SalI site; Chl^R^, Amp^R^. | This work |
| pNF345 | pNF334 with [*aap*(PGR+LPDTG)+DN] inserted in between the XmaI and SalI site; Chl^R^, Amp^R^. | This work |
| pNF350 | *aap*A-lectin allelic exchange vector;  pNF345 with *aap*A-lec cloned directionally into the XmaI site; Chl^R^, Amp^R^. | This work |
| pNF349 | *aap*A-rep+B allelic exchange vector; pNF346 with [*aap*(B+PGR+LPDTG)+DN] inserted in between the XmaI and SalI site; Chl^R^, Amp^R^. | This work |
| pNF342 | *aap*A-lec+B allelic exchange vector;  pNF334 with [*aap*(A-lec+B+PGR+LPDTG)+DN] inserted in between the XmaI and SalI site; Chl^R^, Amp^R^. | This work |

| Bacteriophage | Description | Source or reference |
| --- | --- | --- |
| Φ71 | *S. epidermidis* transducing phage | ([11](#_ENREF_11)) |
| ΦA6C | *S. epidermidis* transducing phage | ([12](#_ENREF_12)) |
| Φ187 | Staphylococcus transducing phage | ([1](#_ENREF_1)) |

Abbreviations: Amp: ampicillin; Tet: tetracycline; Erm: erythromycin; Tmp: trimethroprim;

Chl: chloramphenicol; Kan: kanamycin; R: resistant.

1. Winstel V, Kuhner P, Krismer B, Peschel A, Rohde H. 2015. Transfer of plasmid DNA to clinical coagulase-negative staphylococcal pathogens by using a unique bacteriophage. Appl Environ Microbiol 81:2481-8.

2. Mack D, Siemssen N, Laufs R. 1992. Parallel induction by glucose of adherence and a polysaccharide antigen specific for plastic-adherent *Staphylococcus epidermidis:* evidence for functional relation to intercellular adhesion. Infect Immun 60:2048-57.

3. Handke LD, Slater SR, Conlon KM, O'Donnell ST, Olson ME, Bryant KA, Rupp ME, O'Gara JP, Fey PD. 2007. SigmaB and SarA independently regulate polysaccharide intercellular adhesin production in *Staphylococcus epidermidis*. Can J Microbiol 53:82-91.

4. Schaeffer CR, Woods KM, Longo GM, Kiedrowski MR, Paharik AE, Buttner H, Christner M, Boissy RJ, Horswill AR, Rohde H, Fey PD. 2015. Accumulation-associated protein enhances *Staphylococcus epidermidis* biofilm formation under dynamic conditions and is required for infection in a rat catheter model. Infect Immun 83:214-26.

5. Baba T, Takeuchi F, Kuroda M, Yuzawa H, Aoki K, Oguchi A, Nagai Y, Iwama N, Asano K, Naimi T, Kuroda H, Cui L, Yamamoto K, Hiramatsu K. 2002. Genome and virulence determinants of high virulence community-acquired MRSA. Lancet 359:1819-27.

6. Crosby HA, Schlievert PM, Merriman JA, King JM, Salgado-Pabon W, Horswill AR. 2016. The Staphylococcus aureus Global Regulator MgrA Modulates Clumping and Virulence by Controlling Surface Protein Expression. PLoS Pathog 12:e1005604.

7. Yanisch-Perron C, Vieira J, Messing J. 1985. Improved M13 phage cloning vectors and host strains: nucleotide sequences of the M13mp18 and pUC19 vectors. Gene 33:103-19.

8. Projan SJ, Archer GL. 1989. Mobilization of the relaxable *Staphylococcus aureus* plasmid pC221 by the conjugative plasmid pGO1 involves three pC221 loci. J Bacteriol 171:1841-5.

9. Pang YY, Schwartz J, Thoendel M, Ackermann LW, Horswill AR, Nauseef WM. 2010. agr-Dependent interactions of *Staphylococcus aureus* USA300 with human polymorphonuclear neutrophils. J Innate Immun 2:546-59.

10. Bose JL, Fey PD, Bayles KW. 2013. Genetic tools to enhance the study of gene function and regulation in *Staphylococcus aureus*. Appl Environ Microbiol 79:2218-24.

11. Yajjala VK, Widhelm TJ, Endres JL, Fey PD, Bayles KW. 2016. Generation of a Transposon Mutant Library in *Staphylococcus aureus* and *Staphylococcus epidermidis* Using bursa aurealis. Methods Mol Biol 1373:103-10.

12. Mack D, Bartscht K, Fischer C, Rohde H, de Grahl C, Dobinsky S, Horstkotte MA, Kiel K, Knobloch JK. 2001. Genetic and biochemical analysis of *Staphylococcus epidermidis* biofilm accumulation. Methods Enzymol 336:215-39.
