# Supplementary material for: Glycan-Dependent Corneocyte Adherence of Staphylococcus epidermidis Mediated by the Lectin Subdomain of Aap
Source: mBio. 2021 Jul 13;12(4):e02908-20. doi: 10.1128/mBio.02908-20 (PMC8406310; doi:10.1128/mBio.02908-20)
Supplement: TABLE S2 [file mbio.02908-20-st002.docx]

**Table S2:** Primers used in this study.

| Primer number | Primer name  (F=Forward; R=Reverse) | Sequence (5’–3’)  (Restriction site in bold) | Restriction site; Additional feature |
| --- | --- | --- | --- |
| 2992 | r-*aap*Adom F | CCC**CATATG**GCTGCAGAAGAAAAACAAGTTGAT | NdeI |
| 2993 | r-*aap*Adom R | CCC**CTCGAG***TTA*ACCACTATTACCATTTCCATATTG | XhoI; contains additional 5’ stop codon. |
| 3082 | r-*aap*Arep R | CCC**CTCGAG***TTA*CGTAGTTGGCGGTATATCTATTGT | XhoI; contains additional 5’ stop codon. |
| 2994 | r-*aap*Lec F | CCC**CATATG**GGTAGAGATAATTACGATTTTTACGGT | NdeI |
| 2712 | T7Promoter F | TAATACGACTCACTATAGGG |  |
| 2713 | T7Terminator R | GCTAGTTATTGCTCAGCGG |  |
| 730 | *srtA*UP F | GTCCTAGCCTTTGTATATTGC |  |
| 729 | *srtA*DN R | CAAATTGATTCCCACTCGC |  |
| 889 | *tetM-*ClaI F | AATATCC**ATCGAT**GGTACATGATTACAGATAC | ClaI |
| 862 | *tetM-*ClaI R | AATATCC**ATCGAT**CGATCTCCTCCTTTCCAC | ClaI |
| 3266 | *sdrF*Up F | GGC**GAGCTC**GTTTAGAAAATATTTGGTGTTGAGAAAGGT | SacI |
| 3267 | *sdrF*Up R | ATT**CCCGGG**CCTTGTCTTCTCTTTTTCATACTATACCTC | XmaI |
| 3268 | *sdrF*DN F | GGC**CCCGGG**GCTTTATTTGCAGGTTTAGGAGCATTATTA | XmaI |
| 3269 | *sdrF*DN R | GGC**GTCGAC**GATATATCCAACACCTTCATTATCATAGAA | SalI |
| 3270 | *ermC-*XmaI F | GG**CCCGGG**AATTGAATGAGACATGCTACACC | XmaI |
| 3271 | *ermC*-XmaI R | GG**CCCGGG**AAAACTGGTTTAAGCCGACT | XmaI |
| 3379 | *sdrF*seq F | AGAAAATTCTTTTGATTAAATATAATTTGTTG |  |
| 3380 | *sdrF*seq R | TATAAAGGCAGCATTTTGTCCTAATTTTATTT |  |
| 3200 | *aap*UP F1 | CCC**GAATTC**CCAACAAGAAGTTGCAGGTGC | EcoRI |
| 3201 | *aap*Adom R | CG**CCCGGG**GTATGTACCACTATTACCATTTCCATATTG | XmaI |
| 3202 | *aap*PGR F | CG**CCCGGG**CCAACAAAAGCAGAACCAGG | XmaI |
| 3113 | *aap*DN R | CCC**CTCGAG**GTGACATACGTTTTACCGATGTCA | XhoI |
| 3110 | *aap*UP F | CCC**GAATTC**CCAACAAGAAGTTGCAGGTGCAGA | EcoRI |
| 3130 | *aap*SS R | CG**CCCGGG**TTTCGCTTCATGGCTACTACTTCC | XmaI |
| 3131 | *aap*Bdom F | CG**CCCGGG**GATTTAGATGGTGCAACATTGACA | XmaI |
| 3203 | *aap*Arep R | CG**CCCGGG**CGTAGTTGGCGGTATATCTATTGT | XmaI |
| 3204 | *aap*Alec F | CG**CCCGGG**GGTAGAGATAATTACGATTTTTACGGT | XmaI |
| 3199 | *aap*AlecOut | ACCGTAAAAATCGTAATTATCTCTACC |  |
| 3219 | pJB38seq F | CACCTGACGTCTAAGAAACCA |  |
| 3220 | pJB38seq R | CCTCACATTTGTGCCACCTAA |  |
| 3225 | *aap*UPseq F | CGTTGTTTATGGGGTTCG |  |
| 3226 | *aap*DNseq R | CCCCTCGTTCTTAATGCC |  |
